# Attenuation of inflammatory bowel disease by oral administration of mucoadhesive polydopamine-coated yeast β-glucan via ROS scavenging and gut microbiota regulation

**DOI:** 10.1186/s12951-024-02434-3

**Published:** 2024-04-12

**Authors:** Fan Yang, Yuting Su, Chi Yan, Tianfeng Chen, Peter Chi Keung Cheung

**Affiliations:** 1 School of Life Sciences, The Chinese University of Hong Kong, Shatin, New Territories, Hong Kong China; 2 College of Chemistry and Materials Science, Jinan University, Guangzhou, China

**Keywords:** Yeast β-glucan, Polydopamine, Reactive oxygen species, Adhesiveness, Gut microbiome, Colitis

## Abstract

Treatment for inflammatory bowel disease (IBD) is challenging since current anti-inflammatory and immunosuppressive therapies do not address the underlying causes of the illness, which include increased levels of reactive oxygen species (ROS) and dysbiosis of the gut commensal microbiota. Additionally, these treatments often have systemic off-target effects and adverse side effects. In this study, we have developed a prebiotic yeast β-glucan nanocomplex coated with bio-adhesive polydopamine (YBNs@PDA) to effectively prolong their retention time in the gastrointestinal (GI) tract. The oral administration of YBNs@PDA restored the epithelium barriers, reduced ROS levels, and minimized systemic drug exposure while improved therapeutic efficacy in an acute colitis mouse model. Furthermore, 16S ribosomal RNA genes sequencing demonstrated a higher richness and diversity in gut microflora composition following the treatments. In particular, YBNs@PDA markedly augmented the abundance of *Lachnospiraceae NK4A136* and *Bifidobacterium*, both of which are probiotics with crucial roles in relieving colitis via retaining gut homeostasis. Cumulatively, these results demonstrate that the potential of YBNs@PDA as a novel drug-free, ROS-scavenging and gut microbiota regulation nanoplatform for the treatment of GI disorders.

**Supplementary Information:**

The online version contains supplementary material available at 10.1186/s12951-024-02434-3.

## Introduction

Inflammatory bowel disease (IBD) encompasses a group of autoimmune illnesses characterized by persistent intestinal inflammation [1, 2]. The pathogenesis of the disease remains uncertain, with various factors being implicated, including immune response dysregulation, genetic predisposition, reactive oxygen species (ROS) generation, and gut microbiota dysbiosis [3–5]. The interaction between these factors contributes to the magnitude and duration of IBD, posing significant hurdles to the development of efficacious therapies [6]. Particularly, inflammatory cells infiltrate the intestinal mucosa in individuals with IBD. These cells are responsible for the production and release of significant quantities of cytokines that promote inflammation [7]. Intestinal inflammation and tissue damage are perpetuated by the presence of these cytokines owing to their connection to the over-production of ROS by immune cells [8, 9]. Meanwhile, the gut microbiota can be affected by such chronic intestinal inflammation and ROS generation. ROS eventually generate harmless by-products that act as terminal electron acceptors to promote the growth of facultative anaerobes, leading to gut microbiota dysbiosis [10, 11]. The fecal microbiomes of IBD patients reflect an oxidative stress response with enriched transcription of genes involved in sulfate transport, as well as cysteine and glutathione metabolism [12]. Although modulation of ROS may aid in the restoration of intestinal homeostasis, the GI tract microenvironment is also complicated, requiring local drug release to ensure prolonged efficacy. At present, antioxidant therapies that involve small-molecule drugs have demonstrated restricted efficacy in terms of the durability of ROS modulation and may lead to severe complications (autoimmunity, organ damage, and opportunistic infections) and off-target systemic side effects [13–15]. Thus, alternative methods are highly sought for to break through these limitations for enhancing IBD treatment efficacy.

Recently, the utilization of nanoparticles as drug carriers customized with various ligands to generate amplified localized drug concentrations around IBD lesions is a potential technique to overcome the limitations of conventional drug therapy [16–20]. Polydopamine (PDA) nanoparticles is a melanin-like polymer derived from the oxidative self-polymerization of dopamine monomers [21]. PDA exhibits remarkable redox ability owing to its abundant reductive groups such as catechol and quinone moieties. These properties enable PDA to alleviate oxidative stress by releasing electrons, thereby positioning it a potential ROS scavenger for treating IBD [22]. Additionally, due to its colloidal stability, conductivity, and strong wet adhesion to virtually all types of surfaces that irrespective of the substrate’s chemistry, PDA has extensive applications in surface coating, cell or tissue adhesion, and molecule immobilization [23, 24]. Typically, the catechol groups of PDA, which are abundant in mussel adhesive foot proteins, possess the robust mucosa adhesive properties, resulting in an extended duration of retention within the intestinal tract [25]. Meanwhile, PDA coatings have been observed to possess zwitterionic properties, facilitating nano system penetration of mucus and enhancing cellular uptake by interacting with the positively charged choline groups situated on the epithelial cell membrane [26, 27]. Besides, PDA nanoparticles have the potential to regulate intestinal inflammation through various mechanisms, including the promotion of regulatory T (Treg) cells, inhibition of dendritic cell activation, and suppression of the differentiation of naïve T cells into effector T helper (Th) cells such as Th1, Th2, and Th17 [28]. Hence, the above attributes collectively position PDA as an ideal candidate for developing innovative therapeutic approaches in IBD treatment. Although diminishing exaggerated immune responses that partially caused by ROS is essential in the management of IBD patients, gut microbiota perturbations also promote tissue damage and aberrant host immune responses. As a result, the concomitant utilization of immunogenic suppressant therapy and the manipulation of microbial homeostasis has led to a synergistic suppression of intestinal inflammation [29, 30]. Yeast β-glucan isolated from *Saccharomyces cerevisiae* is a glucose polymer with a linear β (1,3)-glucan backbone with β (1,6)-linked side chains [31]. Extensive research has been conducted on its health benefits, and its biological activity is much higher than that of other β-glucans [32]. In particular, evidence suggests that yeast β-glucans-based treatments may have potential to improve cognition in neurodegenerative disease, attenuate obesity via altering the composition and richness of the gut microbiota [33, 34]. Recently, low molecular weight yeast β-glucans were found to reduce macrophage infiltration, mucosal damage, and colonic inflammation induced by DSS in mice [35]. Although various yeast β-glucan extractions have been utilized to treat IBD and other diseases, there is a paucity of literature on the prophylactic and therapeutic impacts of nanotherapeutics derived from yeast β-glucans on colonic diseases, encompassing immunomodulatory effects and gut microbiota regulation. Therefore, the development of redox-active nanoparticles possesses the capability of both alleviating ROS overproduction and restoring the normal homeostasis of the gut microbiota under environmental with the aim of restructuring a healthy immune microenvironment, represents a promising strategy in IBD therapy.

**Scheme 1:**
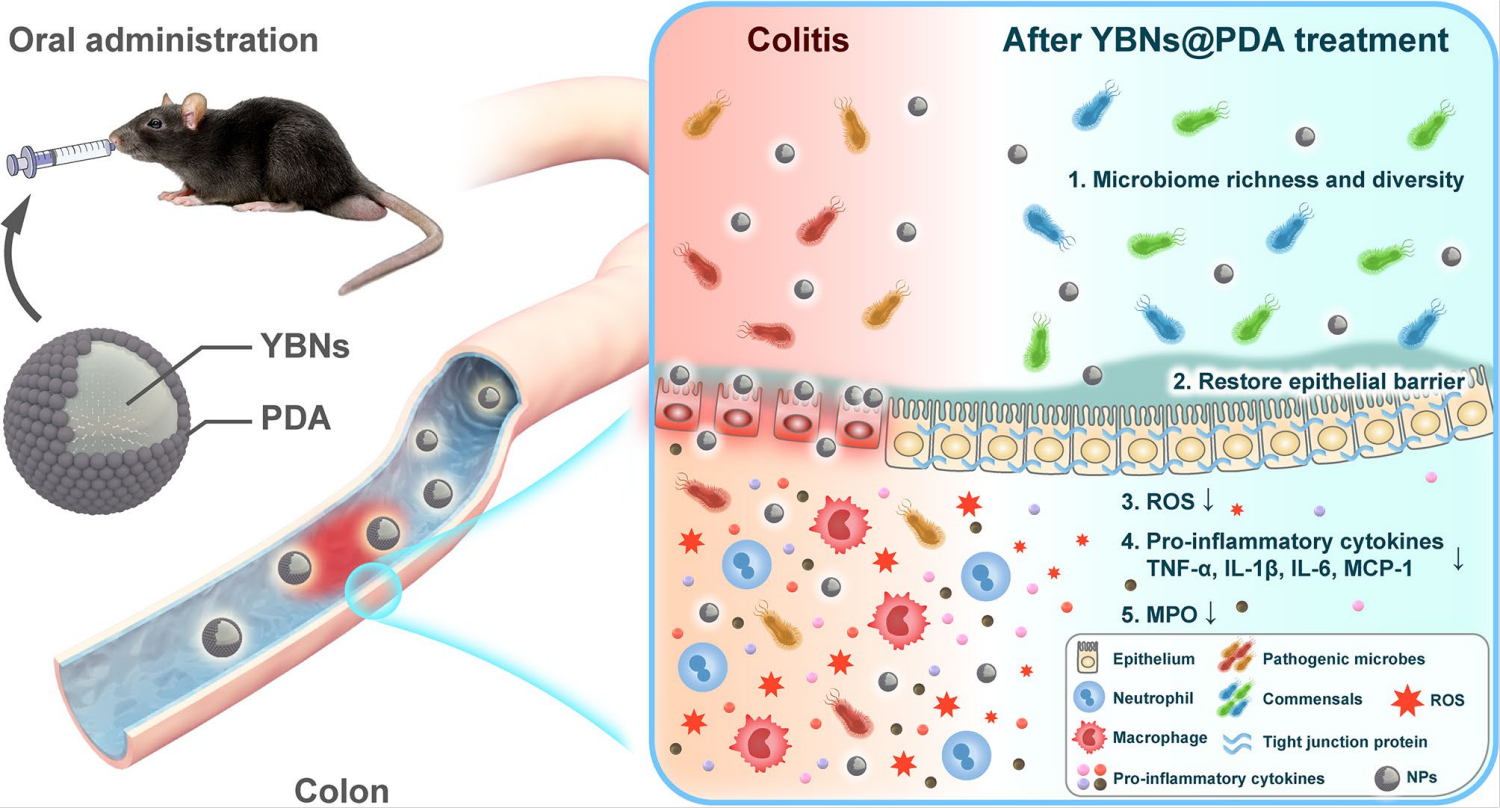
Schematic of the rational design of mucoadhesive YBNs@PDA with ROS-scavenging and gut microbiota regulatory activity for IBD therapy

Herein, we introduce a novel nanotherapeutic system that are composed of nanosized yeast β-glucans coated with polydopamine (YBNs@PDA), which serves to facilitate mucosal healing and alleviate oxidative stress (Scheme 1). The targeted delivery of nano-agents to inflamed colonic tissues can be achieved through the combination of prebiotic and immunomodulatory properties of yeast β-glucans with the polydopamine coating. This approach can effectively supppress ROS levels with minimal collateral damage. Importantly, this platform exhibits an increase in intestinal retention time; increasing the richness and diversity of the gut microbiota; restoring the expression of tight junction-associated proteins; and enhancing intestinal barrier functions in DSS-induced IBD models. Our results suggest that complexation of natural polysaccharide and synthetic polymers as a therapeutic nanoplatform could be a promising approach for the management of various inflammatory conditions.

## Results and discussion

### Synthesis and characterization of YBNs@PDA

**Fig. 1:**
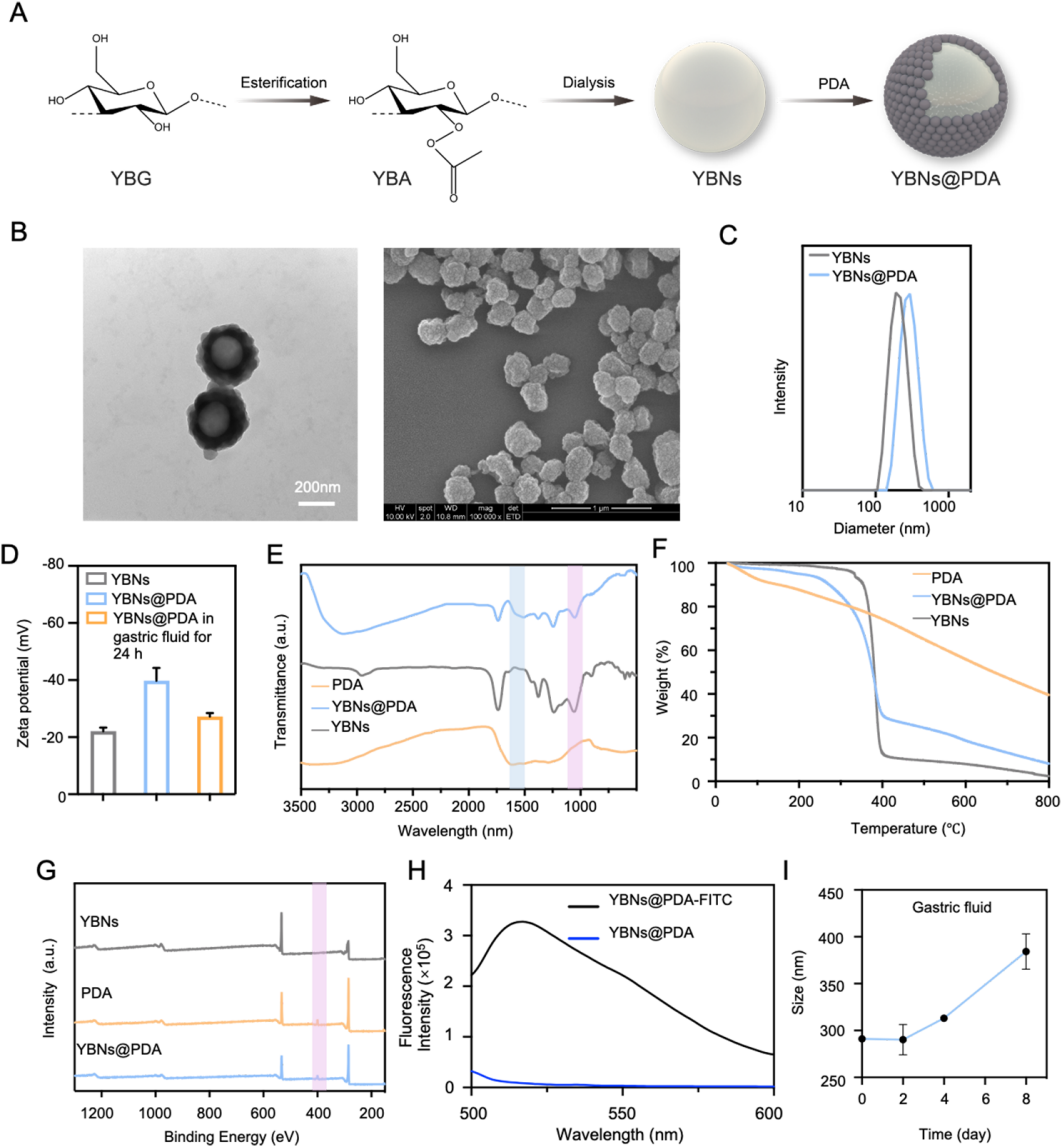
Preparation and characterization of the oral YBNs@PDA. (**A**) Synthesis route of YBNs@PDA. (**B**) TEM and SEM images of YBNs@PDA. (**C**) Size distribution of YBNs and YBNs@PDA. (**D**) Zeta potential of YBNs, YBNs@PDA and YBNs@PDA in gastric fluid for 24 h. (**E**) FT-IR spectrum of PDA, YBNs and YBNs@PDA. (**F**) TGA spectra of YBNs, PDA and YBNs@PDA. (**G**) X-ray photoelectron spectroscopy (XPS) spectra of YBNs, PDA and YBNs@PDA. (**H**) Fluorescence spectra of YBNs@PDA labeled with FITC. (**I**) Hydrodynamic diameter changes of YBNs@PDA in simulated gastric fluid

Generally, YBNs@PDA was prepared by self-assembly of YBNs followed by surface coating with PDA (Fig. 1A; Additional file 1: Fig. S1A). Briefly, yeast β-glucan (YBG) was first modified with formate group as intermediates for the following regioselective derivatization. Subsequently, esterification of yeast β-glucan formate (YBF) was achieved with acetic anhydride addition to form yeast β-glucan acetate (YBA). According to the NMR spectrum (Additional file 1: Fig. S1B), the proton on the formyl group had a distinct chemical shift at approximately 8.3 ppm. Methyl protons on the acetyl group in YBA were responsible for the 1.8–2.2 ppm chemical shift. The structure was further illustrated by Fourier-Transform Infrared spectroscopy (FT-IR) (Additional file 1: Fig. S1C). The bands at around 1750 cm ^-1^, 1430 cm ^-1^, 1370 cm ^-1^ and 1230 cm ^-1^ in the spectra of YBA corresponded to carbonyl C = O stretch vibration, CH_3_ antisymmetric deformation vibration, and carbonyl C-O stretch vibration, respectively. After modifying with ester group, YBA was subsequently dissolved in commonly used organic solvents to prepare homogeneous nanospheres YBNs through nanoprecipitation methods. Next, dopamine hydrochloride was introduced into YBNs solution under an alkaline pH to induce dopamine self-polymerization and form a PDA coating on the surface of YBNs. Transmission Electron Microscopy (TEM) and Scanning Electron Microscope (SEM) (Fig. 1B; Additional file 1: Fig. S2) were initially used to confirm the successful construction of YBNs and YBNs@PDA, revealing homogenous YBNs with distinct PDA shell with a rough surface. To optimize the size of nanoparticles and avoid self-aggregation of PDA, we adjusted the dopamine concentration to prepare YBNs@PDA with a suitable particle size around 300 nm (Fig. 1B; Additional file 1: Fig. S2A). The hydrodynamic size was found to be about 187.1 ± 9.59 nm (polydispersity index (PDI) = 0.09 ± 0.07) for YBNs and 297.4 ± 9.95 nm (PDI = 0.06 ± 0.05) for YBNs@PDA, respectively (Fig. 1C; Additional file 1: Fig. S2B). Zeta potential measurements were also performed to confirm the presence of negatively charged polydopamine on YBNs (Fig. 1D). The successful modification of YBNs@PDA was also proven by FT-IR (Fig. 1E). The N-H bending vibrations, shown by the distinct peaks at approximately 1605 cm^-1^, suggested the existence of indole structures in PDA. The above results were also corroborated by the Thermogravimetric analysis (TGA) and X-ray photoelectron spectroscopy (XPS). Results from TGA performed at temperatures ranging from 100 to 800 °C are shown in Fig. 1F. The weight curve of YBNs@PDA showed a significant decline in contrast to that of the YBNs at approximately 250 °C, which corresponded to the degradation temperature of PDA, confirming the synthesis of PDA coating on YBNs nanoparticles. Since the PDA layer contains amino groups (the source of nitrogen element), XPS analysis (Fig. 1G) showed the existence of the N element in the YBNs@PDA spectrum. The in vivo biodistribution of YBNs@PDA and their cellular interaction were investigated through the utilization of FITC labeling. The successful incorporation of the fluorescein isothiocyanate (FITC) label onto YBNs@PDA was shown by the appearance of an emission peak at 525 nm in the spectra of FITC-labeled YBNs@PDA (Fig. 1H). In addition, the presence of deprotonated hydroxyl groups on PDA facilitated the uniform dispersion of nanoparticles in aqueous solutions, while the size distribution remained unchanged in the presence of serum (Additional file 1: Fig. S2C). These findings indicated that PDA significantly improved the colloidal stability of the nanoparticles and exhibited a better anti-fouling property against serum proteins.

To ensure the stability of orally administered nanoparticles, it is crucial to examine their ability to withstand the harsh conditions of the gastrointestinal tract (GIT) before being absorbed into the body. Hence, particle size alterations were evaluated to determine the stability of YBNs@PDA nanoparticles subsequent to co-incubation with acidic conditions (pH = 1.2), which imitates the gastrointestinal environment [36]. Noticeably, YBNs@PDA showed remarkable stability in acidic gastric environments, as demonstrated in Fig. 1D and I, which illustrates minimal alterations in particle size and zeta potential within 24 h. Therefore, the results demonstrate the remarkable stability of YBNs@PDA and their ability to resist the harsh acidic environment in the GIT, thus promoting their potential as an effective oral drug delivery system.

### ROS scavenging activity in vitro

**Fig. 2:**
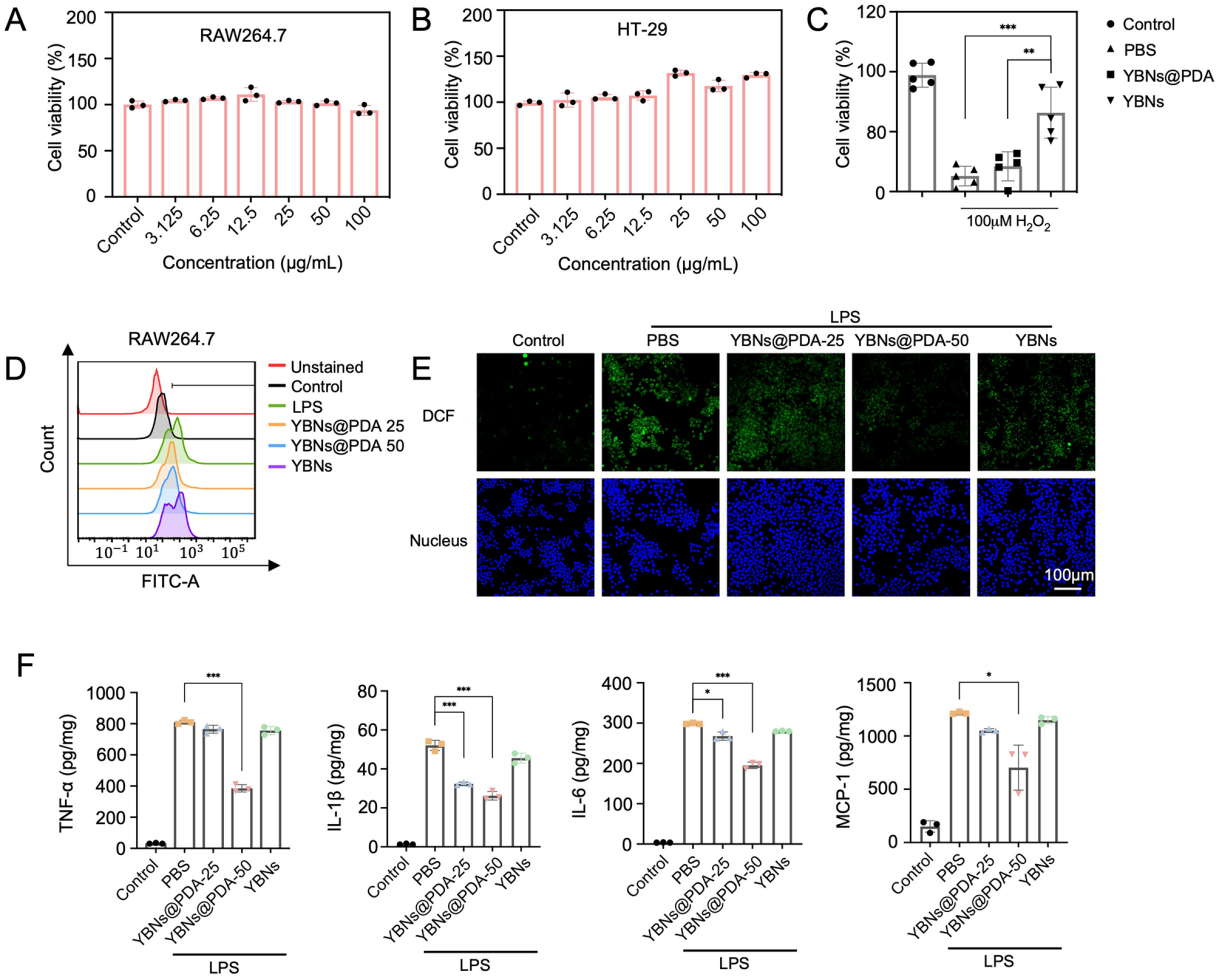
Antioxidant and anti-inflammatory effects of YBNs@PDA. (**A**, **B**) Cytotoxicity profiles of YBNs@PDA towards RAW264.7 and HT-29 cells for 48 h. (**C**) Cell viability of HT-29 treated with YBNs@PDA (50 µg/mL) for 12 h and 100 µM H_2_O_2_ for the following 12 h. (**D**, **E**) Flow cytometry analysis and CLSM images of intracellular ROS levels of RAW264.7 cells after treated with PBS, YBNs@PDA-25 µg/mL, YBNs@PDA-50 µg/mL and YBNs (50 µg/mL). (**F**) Relative expression levels of TNF-α, IL-1β, IL-6 and MCP-1 in LPS-induced RAW 264.7 macrophages after different treatments. Data are presented as mean ± standard deviation from a representative experiment (*n* = 3). *P < 0.05, **P < 0.01, ***P < 0.001, analysed by two-tailed unpaired Student’s t-test

In the inflammatory pathological microenvironments, the extended generation of ROS by infiltrated immune cells (e.g., inflammatory macrophages, dendritic cells and neutrophils) may exacerbate the progression of inflammatory disease through oxidative damage to lipids, proteins, and DNA. Therefore, the inhibition of intracellular ROS and cytoprotective effect by nanoparticles were evaluated. Assessed by MTT assay, we discovered that YBNs@PDA formulations (concentrations ranging from 3.125 to 100 µg/mL) were noncytotoxic to human intestinal epithelial HT-29 and murine macrophage RAW 264.7 cell lines after 48 h (Fig. 2A and B), suggesting that YBNs@PDA is a suitable and safe drug candidate to reach the local inflamed colonic regions. Flow cytometry analysis for detecting nanoparticles internalization efficacy in cells are shown in Additional file 1: Fig. S3. YBNs@PDA were rapidly internalized into cells within only 1 h in both the RAW264.7 and HT-29 cells. Using 2′,7′-dichlorodihydrofluorescein diacetate (DCF) labeling, we were able to quantify the increased amount of intracellular ROS generation in RAW 264.7 cells (Fig. 2D and E) and HT-29 (Additional file 1: Fig. S4) under LPS-induced or H_2_O_2_ stress conditions. YBNs@PDA, but not YBNs alone, substantially attenuated the intracellular ROS over-generation. Nonetheless, it is intriguing that YBNs did not elevate further ROS production. YBNs@PDA exhibited greater ROS-scavenging activity than YBNs while preserving HT-29 colonic epithelial cells from cellular damage mediated by ROS, thereby corroborating the efficacy of ROS elimination (Fig. 2C). In contrast, YBNs did not exhibit ROS-responsiveness and were incapable of preserving HT-29 cells, revealing PDA possesses prominent cytoprotective effect. Subsequently, the anti-inflammation effects of YBNs@PDA were examined by measuring the typical pro-inflammatory cytokine levels, including tumor necrosis factor-alpha (TNF-α), interleukin-1 beta (IL-1β), interleukin-6 (IL-6) and monocyte chemoattractant protein-1 (MCP-1). Figure 2F shows that the LPS-induced generation of pro-inflammatory cytokines was greatly reduced by YBNs@PDA, whereas no noticeable impact was seen in the YBNs-treated group, which was consistent with the findings of the anti-oxidative effects by YBNs.

### Retention of YBNs@PDA in Colon

**Fig. 3:**
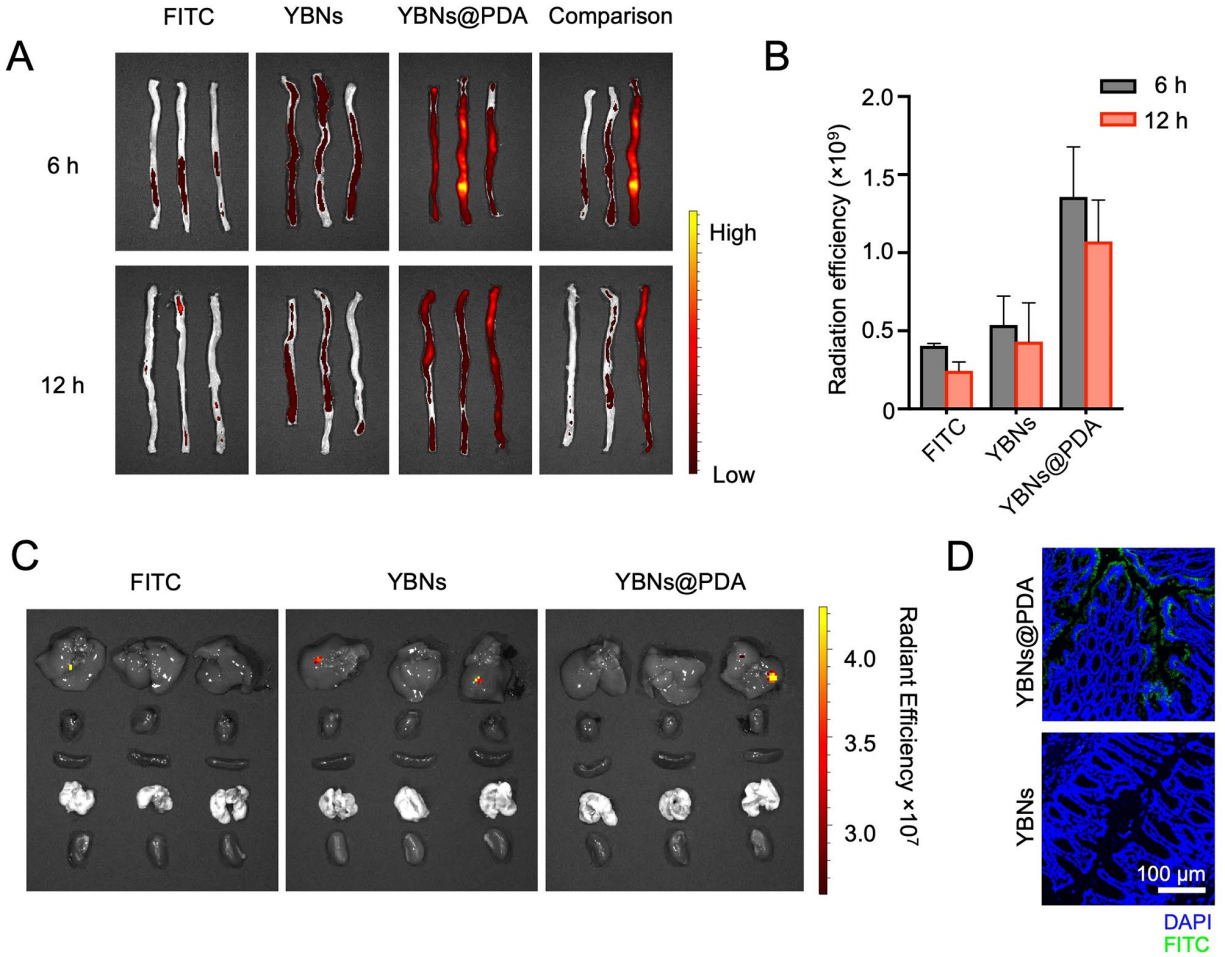
YBNs@PDA localizes in inflamed colon in DSS-treated mice. (**A**, **B**) After 6 h and 12 h of treating animals with 10 mg/kg free-FITC, YBNs-FITC or YBNs@PDA-FITC, colons were imaged by in vivo imaging system (IVIS) (**A**) and quantified for FITC fluorescence signal (**B**). Comparison picture was taken by comparing one colon sample from each group. (**C**) Ex vivo fluorescence images of major organs collected from colitis mice 12 h after oral gavage of free-FITC, YBNs-FITC or YBNs@PDA-FITC, respectively. (**D**) CLSM images sections of the distal colon tissues from mice orally administered with YBNs-FITC or YBNs@PDA-FITC

To determine whether surface coating of mucoadhesive PDA could prolong the retention duration of nano-agents in the gut. The biodistribution and absorption were monitored in mice with acute colitis after oral administration with nanoparticles labeled with fluorescent dye FITC. The real-time monitoring of FITC-conjugated YBNs@PDA was carried out using an in vivo imaging system to ascertain the temporal prediction and site specificity of the nano-agents. Although most of the FITC-YBNs and FITC-YBNs@PDA entered colon after 6 h post-administration as shown in Fig. 3A, the fluorescence intensity observed in the images of FITC-YBNs@PDA was significantly greater than that of FITC-YBNs in the colon at both 6 and 12 h post-administration (Fig. 3B). Consequently, these observations confirmed that the utilization of PDA coating has the potential to enhance the retention time of YBNs@PDA up to 12 h compared with YBNs, thereby preserving the nano-agent concentration in the colon. Based on the fluorescent images, a significant proportion of the nanodrugs were found to be concentrated within the digestive tract. Negligible fluorescence was found in other organs such as the heart, lung, liver, and kidney (Fig. 3C). This phenomenon indicated that there is minimal systemic circulation of YBNs@PDA following oral administration, and that faecal excretion may be the predominant elimination pathway for these nanoparticles.

Further study for the specific location of different nanoparticles was carried out by visualizing colon tissue penetration using Confocal Laser Scanning Microscopy (CLSM). Thus, we dissected the colon from post-administration of FITC-labeled YBNs@PDA and stained it with DAPI for nucleus reveal. As demonstrated in Fig. 3D, an evident green fluorescence signal of FITC-YBNs@PDA was stronger at the lesion site where the signal was barely observed in YBNs treated group. These findings validate the mucoadhesive capability of PDA-modified YBNs nanoparticles for intestinal absorption, allowing the nanoparticles efficiently enter into the intestinal mucosa. PDA-modified nanoparticles benefit from mucus penetration and enhanced intestinal absorption due to the zwitterionic characteristics conferred by the anionic and cationic groups in PDA polymers [26]. Taken together, our findings show that PDA-modified YBN nanoparticles have the potential to serve as effective nano-agents for the purpose of administering oral medication in the future.

### Prophylactic efficacy against acute colitis in a mouse model

**Fig. 4:**
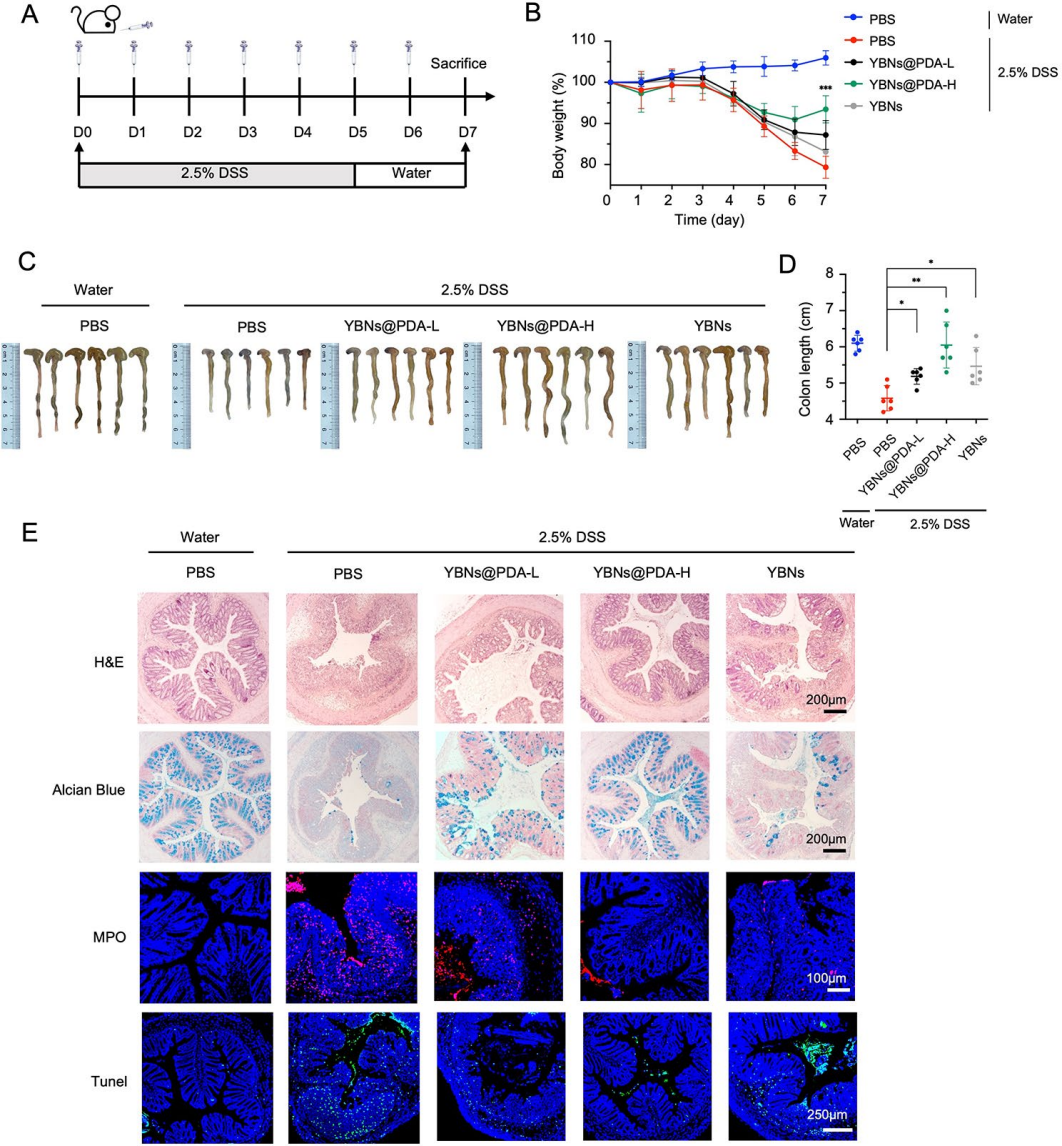
Protective effect of YBNs@PDA in a DSS-induced acute colitis mice model. (**A**) Experimental timeline for different treatments. (**B**) Daily bodyweight changes in each group. YBNs (10 mg/kg) or YBNs@PDA (L: low dose: 5 mg/kg; H: High dose: 10 mg/kg). (**C**) Macroscopic colon appearance of each group. (**D**) Colon length was determined in the indicated groups. (**E**) Representative hematoxylin and eosin (H&E) staining images, alcian blue images, MPO and Tunel images of colon tissue of each group, respectively. Data are presented as mean ± standard deviation from a representative experiment (*n* = 6). *P < 0.05, **P < 0.01, ***P < 0.001, analysed by two-way ANOVA (**B**) and two-tailed unpaired Student’s t-test (**D**)

Based on the above results, YBNs@PDA features several advantages such as good biocompatibility, antioxidant function, inflamed site adhesion and penetration, and anti-inflammation. Next, we sought to determine whether or not YBNs@PDA was effective as a treatment for DSS-induced colitis in a mouse model (Fig. 4A). Acute colitis was induced in 6-week-old mice by administering 2.5% DSS in drinking water for 5 days. At the same time, mice received oral gavages of YBNs@PDA or YBNs for 7 days. Colitis mice orally given PBS served as a negative control. On day 7, all mice were sacrificed to assess colon length, histological severity, IBD-associated colonic myeloperoxidase (MPO) activity, tunnel assays, ROS detection, FISH staining, and protein expressions of tight junction-associated proteins (Occludin and Claudin-1).

Our findings showed that the administration of YBNs@PDA significantly improved prophylactic and therapeutic outcomes in DSS-induced acute colitis. Mice with acute colitis treated with a high dose of YBNs@PDA promptly recovered their bodyweight and disease activity index (DAI) (Fig. 4B and Additional file 1: Fig. S5A). The YBNs@PDA group also had a much longer colon than the other treatment groups, making them equivalent in size to healthy mice (Fig. 4C and D). Furthermore, a histological analysis was conducted to assess the therapeutic efficacy of YBNs@PDA against DSS-induced colitis. Images of H&E staining revealed that the PBS-treated group displayed several IBD symptoms, including immune cell infiltration, goblet cell depletion, and loss of colonic crypts. However, YBNs@PDA treatment could protect colon against those pathological damages (Fig. 4E). We implemented a histological scoring system based on a published literature [37]. Histopathology scores for all DSS-treated groups were considerably higher than those of the healthy control group, suggesting increased inflammatory effects. However, mice administered a high dosage of YBNs@PDA exhibited a substantial reduction in histopathological inflammation (Additional file 1: Fig. S5B), indicating that YBNs@PDA could remarkably promote the repair of colon tissues. Furthermore, goblet cells in the colon tissue treated by high-dose YBNs@PDA were more prominent than in the PBS-treated group, as shown by Alcian blue staining (Fig. 4E). Interestingly, when compared to the PBS group, those administered with YBNs exhibited a slower rate of weight loss, a longer colon, and better histopathological ratings, although this prophylactic efficacy against colitis was not as potent as YBNs@PDA group.

Neutrophil activity in the colon was evaluated by measuring MPO activity in the colon. Colon tissue from colitis control animals showed a significant increase in MPO expression (Fig. 4E), suggesting a high neutrophil infiltration level. All of the treatment groups had lowered MPO levels, among which the high dose YBNs@PDA treatment group had the most pronounced effects overall, dramatically downregulating MPO levels, which indicates the potent anti-inflammatory effect of YBNs@PDA. We further confirm ROS level in the colon using DCF fluorescence imaging. As shown in Additional file 1: Fig. S6A, the elevated intestine ROS levels induced by DSS treatment were reversed with YBNs@PDA intervention, demonstrating the potent ROS-scavenging capability of YBNs@PDA. Excessive production of ROS can trigger inappropriate apoptosis, resulting in colonic damage, intestinal barrier dysfunction, and bacterial translocation [38]. The TUNEL assay confirmed the reduction in apoptosis in the colonic epithelium after YBNs@PDA treatment, which is consistent with the ROS findings.

**Fig. 5:**
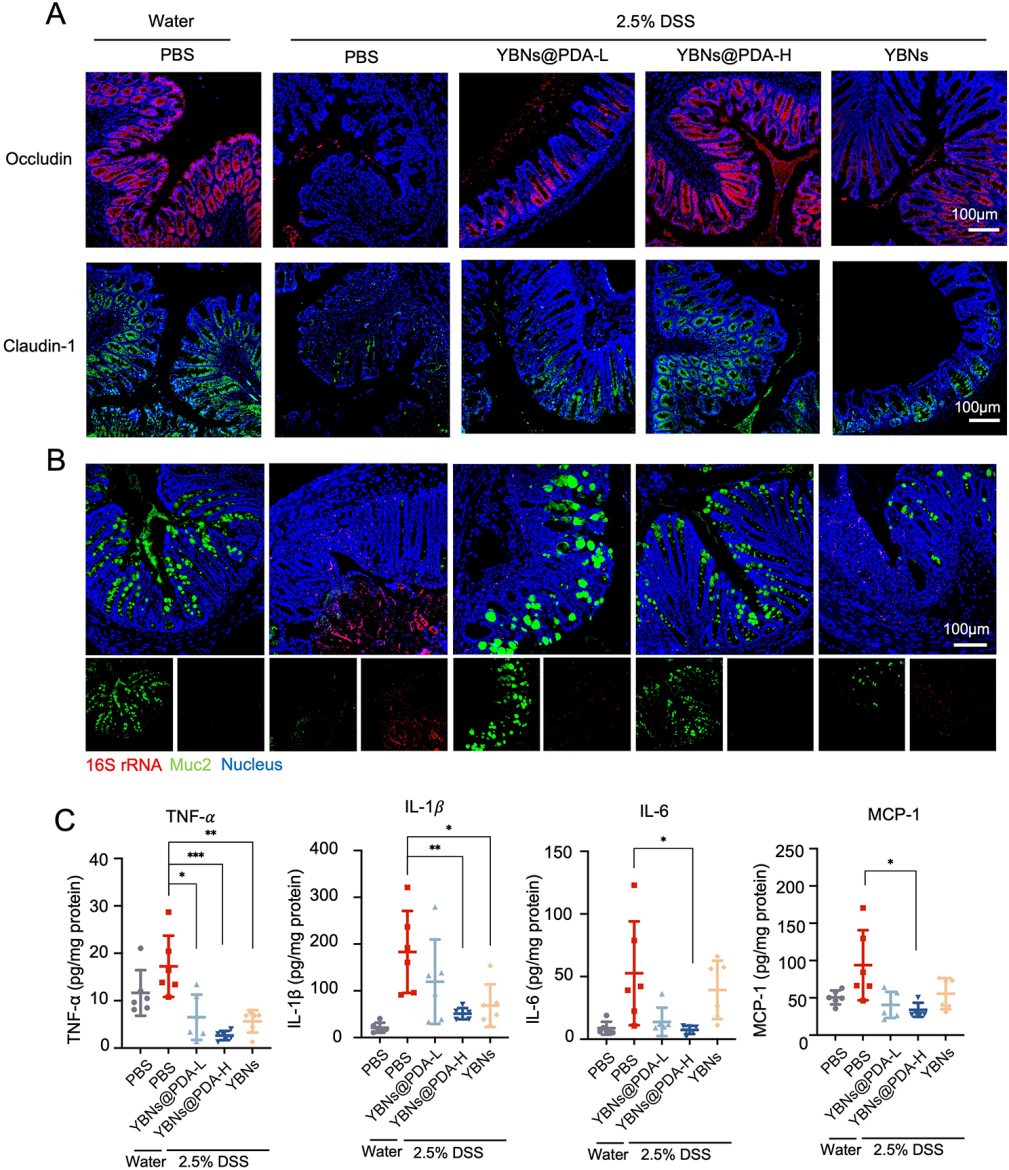
In vivo therapeutic evaluations of YBNs@PDA. (**A**) Immunofluorescence images of occludin and claudin-1 proteins in mice colon tissues. (**B**) Mucus layer and gut bacteria were stained using anti-Muc2 antibody and fluorescent in situ hybridization probe for universal bacterial 16 S rRNA. (**C**) The levels of TNF-α, IL-1β, IL-6 and MCP-1 cytokines in colon tissues from mice treated with different drug formulations were analyzed by ELISA. Data are presented as mean ± standard deviation from a representative experiment (*n* = 6). *P < 0.05, **P < 0.01, ***P < 0.001, analysed by two-tailed unpaired Student’s t-test

IBD is triggered in its earliest stages by oxidative stress caused by an excess of ROS that exert intestinal mucosal barrier disruption [9]. Subsequently, an examination was conducted to determine the impact of YBNs@PDA on the inflammatory state of colonic epithelial cells that have a compromised intestinal barrier function. Tight junction proteins are essential for maintaining normal gut function and gut homeostasis because of the significant functions in maintaining the integrity of the intestinal mucosal barrier and intestinal permeability [39]. Conspicuously, tight junction proteins Occludin and Claudin-1 had their expression patterns restored after being orally supplied YBNs@PDA in DSS-colitis mice (Fig. 5A and Additional file 1: Fig. S6B). Although the expression of Claudin-1 was restored in both the YBNs and YBNs@PDA-treated groups, the latter showed much more efficacy in reversing the damage to the intestinal barrier caused by DSS in mice. The mucus layer acts as a protective barrier against gut microbial penetration of the intestinal mucosal tissue. Due to the erosion of the mucus layer, GI bacteria infiltrate the mucosal tissue of DSS-treated mice to a substantial degree, whereas YBNs@PDA-treated mice exhibited minimal bacterial translocation into the colonic mucosa. (Fig. 5B).

The presence of pro-inflammatory cytokines TNF-α, IL-1β, and IL-6 is a significant predictor of the severity of colitis. They are closely correlated with the production and secretion CD4 + T cells that promote innate and adaptive defense against a variety of pathogens at mucosal tissues [40]. As illustrated in Fig. 5C, those pro-inflammatory factors were less likely to be secreted by colon tissue after YBNs@PDA treatment. The level of MCP-1, a potent activator of monocytes and macrophages, was also decreased after treated with YBNs@PDA. Taken together, the self-assembled YBNs and PDA provided effective protection against colitis in mice.

### Gut microbiome modulation by YBNs@PDA

**Fig. 6:**
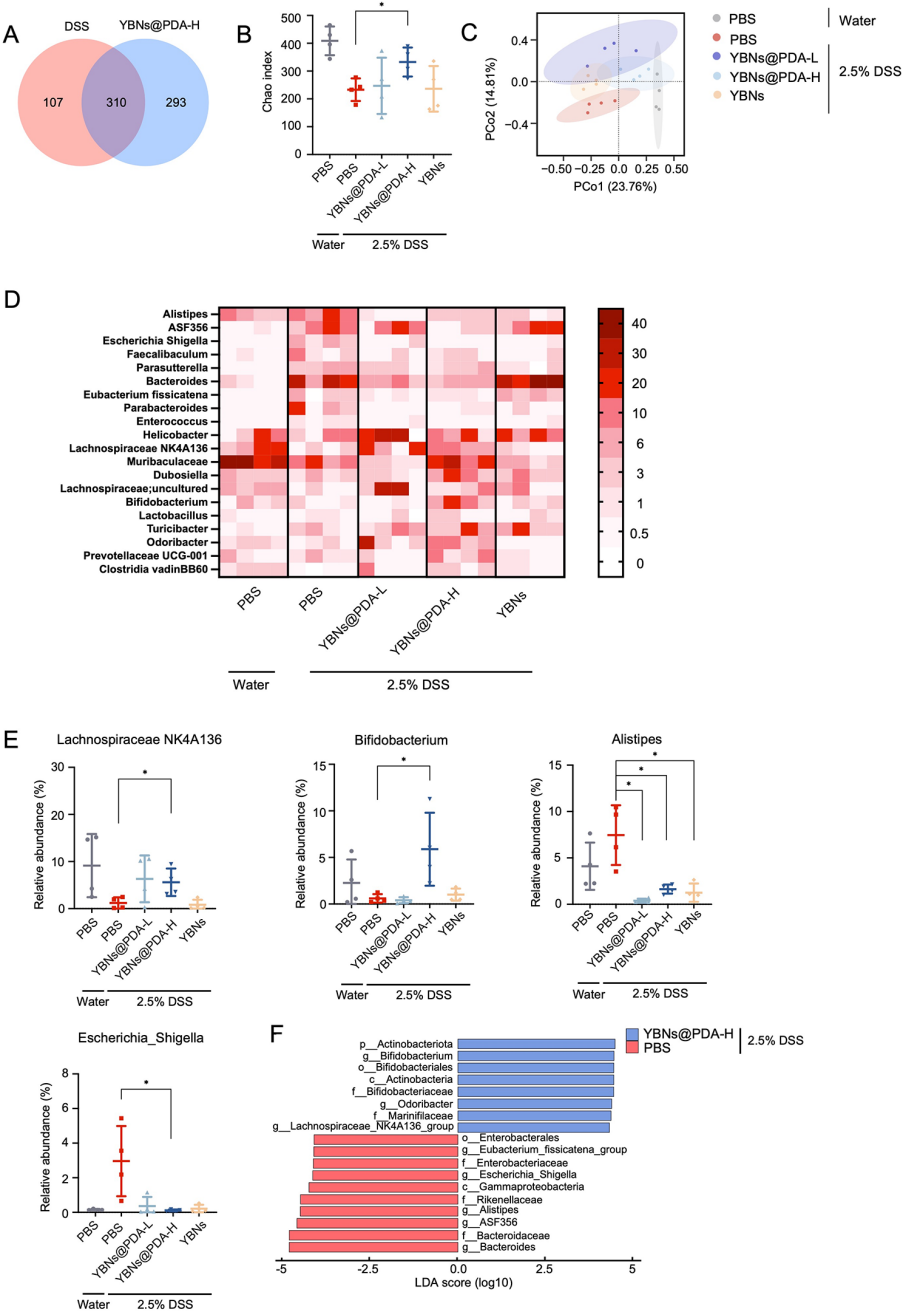
Gut microbiome composition changes with the treatment of YBNs and YBNs@PDA. (**A**) Venn diagram of shared and unique species. (**B**) Alpha diversity was displayed by Chao. (**C**) PCoA plot illustrating the β-diversity according to the Bray-Curtis method. (**D**) Heatmap of the relative abundance of gut microbiome at genus level. The abundance is shown as relative percentage. (**E**) Relative abundance of select taxa at genus level. (**F**) LEfSe analysis of gut microbiota. Data are presented as mean ± standard deviation from a representative experiment (*n* = 4). *P < 0.05, **P < 0.01, ***P < 0.001, analysed by two-tailed unpaired Student’s t-test

Dysbiosis of gut microbiota, which is associated with a range of inflammatory and immune-related conditions, is a contributing factor to the persistence of mucosal inflammation, thereby promoting the IBD progression. After confirming the therapeutic effectiveness of YBNs@PDA in treating colitis, we sought to investigate whether YBNs@PDA would have an impact on gut microbiota regulation. We characterized the microbial composition of fecal samples collected from healthy and colitic mice after treatment with PBS, YBNs, and YBNs@PDA. By sequencing the V3-V4 region of 16S ribosomal RNA gene, we may learn about the diversity of bacteria that cause colitis in mice. Significantly, YBNs@PDA enhanced the richness of the gut microbes compared to the DSS group, as indicated by Venn diagram analysis and the high values of the Chao index (Fig. 6A and B). Notably, PCoA suggested that YBNs and YBNs@PDA-treated mice exhibited a drastically different microbiota composition than in PBS-treated colitis animals (Fig. 6C), confirming that YBNs also tend to regulate dysbiosis. The gut microbiome of the high dose YBNs@PDA treated group exhibited similarities to the faecal communities of healthy mice, in comparison to the other treatment groups. This finding is consistent with the prophylactic efficacy of YBNs@PDA against IBD. When focusing on the genus level, YBNs@PDA treatment remarkably enhanced the abundance of butyrate producing bacteria *Lachnospiraceae NK4A136* [41] and probiotics *Bifidobacterium* [42] (Fig. 6D and E), and dramatically down-regulated colitogenic bacteria such as *Alistipes* and *Escherichia_shigella* [43, 44]. Both phenomena are symptomatic of a dysbiotic microbiota that has been indicated in IBD. To further identify the biologically informative features that help in differentiating microbiome composition from different groups, linear discriminant analysis (LDA) effect size (LEfSe) analysis was applied (Fig. 6F). In line with the above findings, *Lachnospiraceae NK4A136*, *Odoribacter*, *Marinifilaceae* and *Actinobacteriota*, was predominant in YBNs@PDA treated group, whereas PBS treated group showed an enrichment of IBD-associated *Escherichia shigella*, *Bacteroides*, *Enterobacterales*, ASF356, *Eubacterium_fissicatena*, *Alistipes* and *Gammaproteobacteria* pathogens (LDA (log_10_) > 4.0, *P* < 0.05). To investigate the relationship between gut microbiota and gut immunity in colitis under YBNs@PDA administration, a correlation analysis was conducted utilizing the Spearman’s rank correlation method. The study found a significant correlation between the production of pro-inflammatory cytokines, specifically TNF-α and IL-6, and the presence of the beneficial *Lachnospiraceae NK4A136*. Conversely, a negative correlation was observed between the production of pro-inflammatory cytokines and the presence of pathogenic bacteria (Additional file 1: Fig. S7).

### Moderation of colitis in a delay setting of treatment

**Fig. 7:**
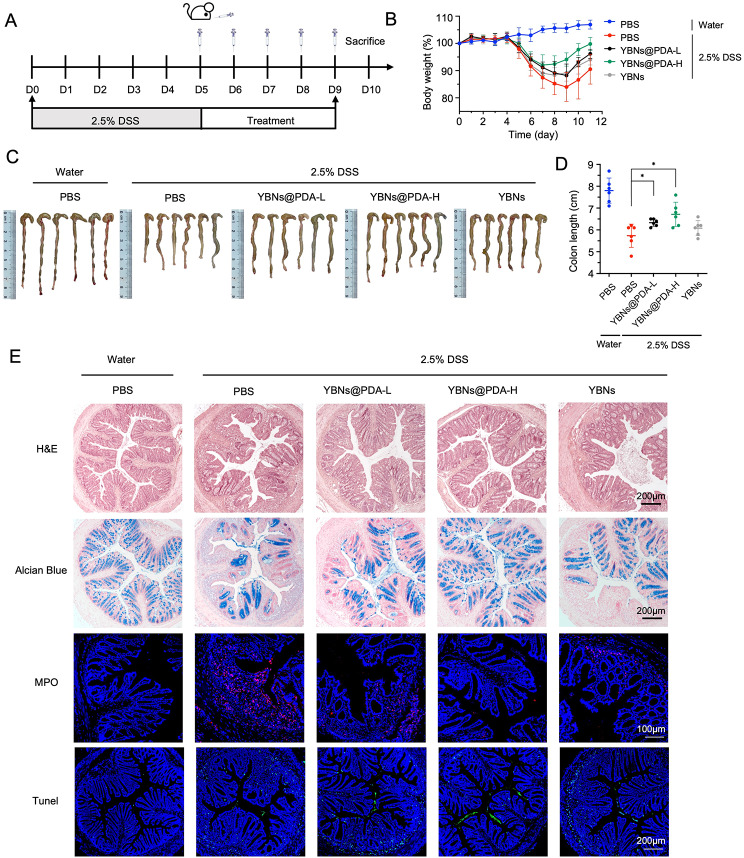
Colitis alleviation effect of YBNs@PDA in a delayed therapeutic setting. (**A**) Experimental timeline for different treatments. (**B**) Daily bodyweight changes in each group. YBNs (10 mg/kg) or YBNs@PDA (L: low dose: 5 mg/kg; H: High dose: 10 mg/kg). (**C**) Macroscopic colon appearance of each group. (**D**) Colon length was determined in the indicated groups. (**E**) Representative hematoxylin and eosin (H&E) staining images, alcian blue images, colonic MPO and Tunel images of colon tissue of each group, respectively. Data are presented as mean ± standard deviation from a representative experiment (*n* = 6). *P < 0.05, analysed by two-tailed unpaired Student’s t-test (**D**)

**Fig. 8:**
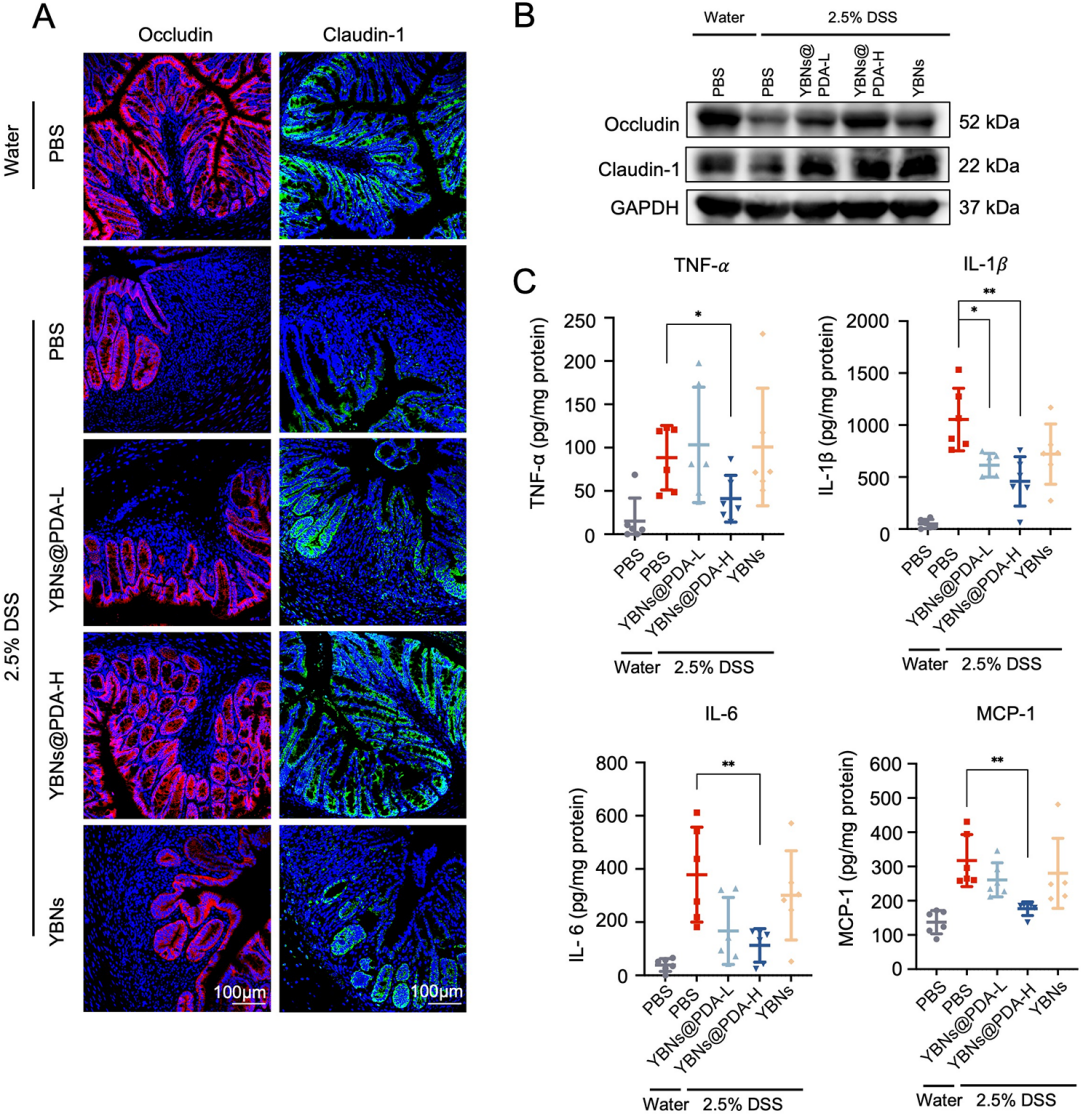
In vivo therapeutic evaluations of YBNs@PDA. (**A**) Immunofluorescence images of occludin and claudin-1 proteins in mice colon tissues. (**B**) Western blot analysis of occludin and claudin-1 proteins in mice colon tissues. (**C**) The levels of TNF-α, IL-1β, IL-6 and MCP-1 cytokines in colon tissues from mice treated with different drug formulations were analyzed by ELISA. Data are presented as mean ± standard deviation from a representative experiment (*n* = 6). *P < 0.05, **P < 0.01, analysed by two-tailed unpaired Student’s t-test

Lastly, we assessed the therapeutic potential of YBNs@PDA when administered at a later stage of disease progression in mice with acute colitis. Mice were initially treated with 2.5% DSS added in the water for 5 days to induce colitis, and randomly divided into four groups: PBS, YBNs and YBNs@PDA (two dosages) (Fig. 7A). On day 8 of the experiment, oral administration with YBNs@PDA showed an earlier recovery in body weight than other treatment groups (Fig. 7B). Besides, YBNs@PDA treatment potently protected colon length from DSS-induced shortening and lowered the DAI scores, indicating a significant decreased colonic inflammation in mice (Fig. 7C and D and Additional file 1: Fig. S8). Further, as demonstrated by H&E, Alcian blue staining, and MPO expression, YBNs@PDA treatment significantly reduced tissue injury and histological inflammation in colon tissue of mice (Fig. 7E). From the TUNEL assay images shown in Fig. 7E, after treatment with a high dose of YBNs@PDA, the number of apoptotic cells in the epithelium has been observed to decrease significantly. Representative immune-fluorescence images and western blot show increased occludin and claudin-1 at the colitis epithelium with YBNs@PDA group compared to PBS treated group (Fig. 8A, B and Additional file 1: Fig.S9). Additionally, YBNs@PDA treatment also suppressed the levels of pro-inflammatory cytokines such as TNF-α, IL-1β, IL-6 and MCP-1 in colon (Fig. 8C).

### In vivo safety of YBNs@PDA

Following the successful therapeutic results achieved by YBNs@PDA in a colitic mouse model, we then assessed the biosafety of YBNs@PDA and YBNs in a murine model, since the systemic biosafety of nanomaterials-based drugs in vivo is also a crucial factor for their clinical application. Six-week-old male C57BL/6J mice were randomly divided into four groups: normal group, YBNs group and YBNs@PDA groups with two doses, to receive treatments for 7 days. As expected, body weights exhibited nearly no difference as compared across all treatment groups (Additional file 1: Fig. S10B). We could not identify any obvious symptoms of systemic toxicity and necrosis in the major organs from the histological investigation of the YBNs and YBNs@PDA-treated group (Additional file 1: Fig. S10A). Alanine aminotransferase (ALT), aspartate aminotransferase (AST), blood urea nitrogen (BUN) and creatinine (CRE) belong to liver and kidney function parameters, all of which were performing in the normal range (Additional file 1: Fig. S10C). These results suggest excellent biosafety of the prepared YBNs@PDA and YBNs.

## Conclusion

Given the fact that multiple sources of ROS in the context of microbes necessitate the presence of crucial regulatory mechanisms to prevent the potential disorder that could occur during severe inflammation. To this end, we have developed a multifunctional platform of YBNs@PDA to safeguard colon tissues from the deleterious impacts associated with IBD. Owing to the mucoadhesive PDA layer that that allows YBNs attach to inflamed colon mucosa, this nano-agents prolonged the retention duration in the intestine, enabling them to maximize the drug bioavailability. The core YBNs showed greater potential to achieve positive gut microbiota regulation when coated with the PDA layer on their surface. The synergistic effect of YBNs@PDA on potently scavenging ROS and modifying microbial homeostasis in intestinal microenvironments simultaneously, resulted in considerably increased protective and therapeutic efficacy in an acute colitis mouse model. Body weight loss and colon length decrease driven by DSS-induced acute colitis were markedly reduced by YBNs@PDA. Moreover, disease severity in colitic mice receiving high dose of YBNs@PDA decreased significantly. The gut microbiota of DSS mice treated with YBNs@PDA was altered considerably compared to PBS-treated mice, demonstrating the capacity to stimulate the colonization of bacteria that produce short-chain fatty acids and favourably regulate the gut microbiota. It is worth noting that PDA nanoparticles may undergo self-degradation after completely reacting with ROS or being digested by microbes [21]. Hence, it is plausible to manage acute colitis by combining strategies that aim to restore gut microbiota and ROS elimination with strategies that aim to restore gut immunological homeostasis.

## Methods and materials

**Materials**. Yeast β-glucan were extracted from baker yeast (Angel, China). Dextran sodium sulfate (36–50 kDa) was obtained from MP Biomedical. Thiazolyl blue tetrazolium bromide (MTT), Dimethyl-sulfoxide (DMSO) and Dopamine hydrochloride, FITC and LPS were purchased from Sigma-Aldrich Co. (St. Louis, MO, USA). Spectra/Por membrane was purchased from Spectrum Laboratories Inc. (CA, USA). Penicillin-Streptomycin (P.S.), fetal bovine serum (FBS), Dulbecco’s modified eagle medium (DMEM) and trypsin-EDTA were purchased from Gibco (Karlsruhe, Germany). DCF, ELISA kits (TNF-α, IL-1β, IL-6, MCP-1), Claudin-1antibody (374,900), Alexa fluor 647 goat anti-rabbit IGG (A21245) were purchased from Thermo Fisher Scientific (Carlsbad, CA, USA). Occludin antibody (AB216327), Myeloperoxidase antibody (AB208670), Mucin-2 antibody (AB272692), Alexa fluor 488 rabbit anti-mouse IGG H&L (AB150125), Alexa fluor 488 goat anti-rabbit IGG H&L (AB150077) were purchased from Abcam (Chicago, IL, USA). TUNEL kit and Antifade Mounting Medium with DAPI were obtained from Beyotime Biotechnology (Shanghai, China).

**Synthesis of YBNs@PDA.** YBNs was synthesized according to the previous report with slight changes [45]. Briefly, water- and alkaline-soluble yeast β-glucan fractions were extracted and freeze-dried. The extracted yeast β-glucan was added into formic acid, and the mixture was heated to 85 °C for 3 h. The reaction mixture was subsequently filtered and the filtrate was mixed with 300 mL ethanol to precipitate the product YBF which was lyophilized after being rinsed twice with ethanol. YBF was then re-dissolved in pyridine and stirred for 1 h at 85 °C, after which cooled acetic anhydride was added dropwise and the mixture was allowed to react for 6 h. To produce yeast β-glucan ester (YBA), the mixture was filtered, isolated by precipitation in ethanol, boiled for 3 h for de-formylation, washed twice with ethanol, and dried by lyophilization. YBNs nanoparticles was prepared by nanoprecipitation. Briefly, YBA was added to acetone, followed by the same volume of distilled water added dropwise to precipitate the nanoparticles which were dialyzed against distilled water. YBNs were then dispersed in 10 mM Tris buffer with pH = 8.5. Certain amount of dopamine hydrochloride was first dissolved in ddH_2_O, and subsequently added into the YBNs dispersion to achieve the final concentration of dopamine hydrochloride 0.4 mg/mL. This reaction was carried out by stirring for 3 h. The final product was centrifuged and washed by water for three times.

**Physicochemical characterization of YBNs@PDA.** Particle sizes, polydispersity index (PDI), and ζ-potentials of PDA@YBNs and YBNs were evaluated using a Malvern Zetasizer Nano ZS. The morphology of YBNs was studied by both transmission electron microscopy (TEM) (Hitachi H-7650, Japan) and scanning electron microscopy (SEM) (JEOL JSM-7800 F). YBNs, PDA and YBNs@PDA samples prepared in KBr disc were scanned by a Fourier Transform Infrared (FT-IR) spectrometer (Spectrum One, PerkinElmer, USA) and a fluorescent spectrometer (Shimadzu RF 6000). The XPS fingerprint of the nanoparticles was obtained by X-Ray Photoelectron Spectroscopy (XPS) (Thermo Fisher Nexsa). The YBNs, PDA, and YBNs@PDA were subjected to thermogravimetric analysis (TGA) using a TGA6 (Perkin Elmer) analyser with a sample size of 5 mg heated in a nitrogen environment from 0 to 800 °C at a heating rate of 10 °C per minute.

**Cellular uptake.** For cellular uptake of nanoparticles, 6 or 12 well plates were seeded with RAW264.7 or HT-29 cells and incubated overnight at 37 °C. The cells were then treated with nanoparticles or free FITC in a fresh complete DMEM for different treatment times. Flow cytometry (BD FACSVerse) or a Zeiss microscope was used to quantitatively analyze the FITC intensity levels in the cells.

**Intracellular ROS measurement and in vitro anti-inflammatory activity.** 2′,7′-dichlorodihydrofluorescein diacetate (DCF), a fluorescent probe for ROS, was used in flow cytometry and confocal laser scanning microscopy (CLSM) study to determine intracellular ROS levels. RAW264.7 and HT-29 cells were initially treated with YBNs or YBNs@PDA (50 µg/mL) for 12 h. Both LPS at 0.1 mg/mL and H_2_O_2_ at 100 µM were used to activate the cells for 2 h. At the end, DCF was applied for the ROS staining according to the manufacturer’s instructions. RAW264.7 macrophages were treated with different nanoparticles in 12 or 24-well plates to assess their anti-inflammatory efficacy in vitro. Following 24 h of incubation, cells were stimulated with 0.1 mg/mL LPS for 6 h. The supernatants were then collected, and the levels of several inflammatory factors including TNF-α, IL-1β, IL-6, and MCP-1 were determined using ELISA kits according to the manufacturer’s instructions.

**Biodistribution Study.** Mice with DSS-induced acute colitis were allowed to fast for 24 h while still having access to water in order to compare colon absorption of FITC-YBNs and FITC-YBNs@PDA. Subsequently, mice were given an oral gavage of each sample with 10 mg kg^-1^ for either 6–12 h, followed by being sacrificed for colon collection. The PerkinElmer IVIS Lumina III In Vivo Imaging System was used to assess the fluorescence signal. After 12–24 h of fixation in 4% paraformaldehyde, colons were embedded in OCT and sectioned using a microtome. After that, slides were mounted and observed using a Leica TCS SP8 Confocal Microscope.

**DSS-induced mouse models of colitis.** All animal work was carried out in accordance with the regulations of the Animal Experimentation Ethics Committee of The Chinese University of Hong Kong (Ref. 22-177-MIS). C57BL/6J mice (male, aged 6 weeks) were given 2.5% DSS in their drinking water for 5 days before being switched to normal water in the treatment duration. PBS was orally given to healthy mice as a normal control group. Colitis mice were administered YBNs orally at dosages of 10 mg kg^-1^ and YBNs@PDA at doses of 5 mg kg^-1^ or 10 mg kg^-1^ for 5 or 7 days. Mice with colitis that were treated with PBS served as a negative control. During the whole experiment, all mice had free access to water and a regular laboratory diet. The day after the last treatment, all mice were sacrificed and their entire colons were removed for further assessment such as colon length assessment, H&E staining, and other histopathological analysis.

**Histopathology study.** Colon tissues or organs fixed in 10% formaldehyde solution underwent tissue processing before paraffin embedding. The paraffin sections of 5 μm were cut by a Leica microtome, stained for H&E, and dried overnight. The H&E images were taken with a Zeiss microscope. Alcian blue was used to stain acid mucosubstances and acetic mucins such as GAGs.

**Immunofluorescence.** Sections of paraffin-embedded colon samples were deparaffinized and rehydrated. Sodium citrate buffer was utilized to recover the antigens, followed by permeabilization with 0.25% Triton X-100 for 15 min, incubating with 0.3% H_2_O_2_ and blocking with 5% goat serum. The sections were then treated with the primary antibodies (Occludin, Claudin-1, MPO) at 4 °C for one night, followed by secondary antibodies incubation for 1 h at room temperature. After being rinsed in PBS, the sections were mounted in antifade mounting media containing DAPI. Confocal microscopy using a Leica TCS SP8 was used to capture the fluorescent pictures.

**Terminal deoxynucleotidyl transferase-mediated dUTP nick end labeling (TUNEL) staining.** The TUNEL assay was performed in accordance with the manufacturer’s instructions. Briefly, xylene was used to deparaffinize tissue sections of the colon, and PBS was used to rehydrate the sections. The sections were then treated with proteinase K and lasted for 20 min at 37 °C. The sections were washed three times in PBS buffer and then treated with Labeling Mix and TdT Enzyme for 1 h in the dark at 37 °C, followed by rinsing with PBS to remove any remaining debris. Finally, a Leica TCS SP8 Confocal Microscope was used to examine the DAPI-stained mounted slides.

**Fluorescence in situ hybridization (FISH) of 16 S rRNA.** FISH analysis was performed via a Cy3-labeled Eubacterial (universal) 16 S rRNA probe (Ribo technologies) to identify bacterial colonization of the colonic mucosa. Briefly, paraffin-embedded colon sections were deparaffinized, stained with a hybridization mixture comprising a Cy3-conjugated-eubacterial 16 S rRNA probe overnight in the dark at 50 °C. Sections were washed with PBS and stained with anti-Muc2 antibody for mucin staining. Secondary antibody anti-rabbit-488 was applied, followed by DAPI staining to reveal nucleus. Leica TCS SP8 Confocal Microscope was used for the observation of the pictures.

**Gut microbiota 16 S rRNA genes sequencing**. After receiving various treatments, colon contents were collected, frozen in liquid nitrogen, and afterward subjected to DNA extraction. The 16 S rRNA genes (V3 - V4 hypervariable regions) were targeted using PCR amplification and then sequenced on the DNBSEQ PE300 technology (BGI Tech, China). QIIME 2 (version 2021.8) was used to analyze the 16 S rRNA gene sequences. DADA2 was used to process the raw read with default settings, assemble and demultiplex the paired-end sequenced dataset to yield amplicon sequence variation (ASV). Then, taxonomic annotation was performed by reference to pre-trained Silva 138 database. Chao metrics were used to determine alpha diversity, while the Bray-Curtis technique was used to determine beta diversity. After QIIME 2 data export, a heatmap was created to display the relative abundance of each group at the genus level. In order to discover taxa that were differentially abundant across biological circumstances of interest, a Linear discriminant analysis effect size (LDA-LEfSe) study was conducted using the online Galaxy web program (http://huttenhower.sph.harvard.edu/galaxy/).

**In vivo toxicity test.** Healthy mice were orally administered with 10 mg kg^− 1^ YBNs, 5 mg kg^− 1^ YBNs@PDA, 10 mg kg^− 1^ YBNs@PDA or PBS for 7 days. Body weight changes were monitored during treatment. Mice serum was collected for biochemistry assessment with several blood parameters: ALT, AST, CRE and BUN. Histological changes of major organs (heart, liver, spleen, lung, kidney and colon) were analysed through H&E staining.

**Statistical analysis**. All measurements were taken with separate samples rather than with repeated measurements. For data analysis, GraphPad Prism version 8 (GraphPad Software; San Diego, CA) was utilized. Unless otherwise specified, statistical analysis was performed using the two-tailed student’s t-test, and results are presented as means ± standard deviations. Statistics were considered to be significant when the P value was less than 0.05.

### Electronic supplementary material

Below is the link to the electronic supplementary material.

Supplementary Material 1

Supplementary Material 2

## Data Availability

The datasets used and/or analysed during the current study are available from the corresponding author on reasonable request.

## Declarations

### Authors’ information

School of Life Sciences, The Chinese University of Hong Kong, Shatin, New Territories, Hong Kong, China.

Fan Yang, Yuting Su, Chi Yan, Peter Chi Keung Cheung.

College of Chemistry and Materials Science, Jinan University, Guangzhou, China.

Tianfeng Chen.

## References

[1] Khor B, Gardet A, Xavier RJ (2011). Genetics and pathogenesis of inflammatory bowel disease. Nature.

[2] Graham DB, Xavier RJ (2020). Pathway paradigms revealed from the genetics of inflammatory bowel disease. Nature.

[3] Caruso R, Lo BC, Nunez G (2020). Host-microbiota interactions in inflammatory bowel disease. Nat Rev Immunol.

[4] Taylor CT, Colgan SP (2017). Regulation of immunity and inflammation by hypoxia in immunological niches. Nat Rev Immunol.

[5] Maloy KJ, Powrie F (2011). Intestinal homeostasis and its breakdown in inflammatory bowel disease. Nature.

[6] Xavier RJ, Podolsky DK (2007). Unravelling the pathogenesis of inflammatory bowel disease. Nature.

[7] Zaiatz Bittencourt V, Jones F, Doherty G, Ryan EJ (2021). Targeting Immune Cell Metabolism in the treatment of inflammatory bowel disease. Inflamm Bowel Dis.

[8] Bourgonje AR, Feelisch M, Faber KN, Pasch A, Dijkstra G, van Goor H (2020). Oxidative stress and redox-modulating therapeutics in inflammatory bowel disease. Trends Mol Med.

[9] Campbell EL, Colgan SP (2019). Control and dysregulation of redox signalling in the gastrointestinal tract. Nat Rev Gastroenterol Hepatol.

[10] Ballal SA, Veiga P, Fenn K, Michaud M, Kim JH, Gallini CA, Glickman JN, Quere G, Garault P, Beal C (2015). Host lysozyme-mediated lysis of Lactococcus lactis facilitates delivery of colitis-attenuating superoxide dismutase to inflamed colons. Proc Natl Acad Sci USA.

[11] Liu J, Wang Y, Heelan WJ, Chen Y, Li Z, Hu Q (2022). Mucoadhesive probiotic backpacks with ROS nanoscavengers enhance the bacteriotherapy for inflammatory bowel diseases. Sci Adv.

[12] Morgan XC, Tickle TL, Sokol H, Gevers D, Devaney KL, Ward DV, Reyes JA, Shah SA, LeLeiko N, Snapper SB (2012). Dysfunction of the intestinal microbiome in inflammatory bowel disease and treatment. Genome Biol.

[13] Moura FA, de Andrade KQ, Dos Santos JCF, Araujo ORP, Goulart MOF (2015). Antioxidant therapy for treatment of inflammatory bowel disease: does it work?. Redox Biol.

[14] Stallmach A, Hagel S, Bruns T (2010). Adverse effects of biologics used for treating IBD. Best Pract Res Clin Gastroenterol.

[15] Bernstein CN, Fried M, Krabshuis JH, Cohen H, Eliakim R, Fedail S, Gearry R, Goh KL, Hamid S, Khan AG (2010). World Gastroenterology Organization Practice Guidelines for the diagnosis and management of IBD in 2010. Inflamm Bowel Dis.

[16] Lee Y, Sugihara K, Gillilland MG, Jon S, Kamada N, Moon JJ (2020). Hyaluronic acid-bilirubin nanomedicine for targeted modulation of dysregulated intestinal barrier, microbiome and immune responses in colitis. Nat Mater.

[17] Wang R, Cao S, Bashir MEH, Hesser LA, Su Y, Hong SMC, Thompson A, Culleen E, Sabados M, Dylla NP (2023). Treatment of peanut allergy and colitis in mice via the intestinal release of butyrate from polymeric micelles. Nat Biomed Eng.

[18] Lamprecht A (2015). Nanomedicines in gastroenterology and hepatology. Nat Rev Gastroenterol Hepatol.

[19] Yang H, Zhu CH, Yuan WL, Wei X, Liu C, Huang JR, Yuan M, Wu YJ, Ling QJ, Hoffmann PR et al. Mannose-rich oligosaccharides-functionalized selenium nanoparticles mediates macrophage reprogramming and inflammation resolution in ulcerative colitis. Chem Eng J 2022; 435.

[20] Shi C, Dawulieti J, Shi F, Yang C, Qin Q, Shi T, Wang L, Hu H, Sun M, Ren L (2022). A nanoparticulate dual scavenger for targeted therapy of inflammatory bowel disease. Sci Adv.

[21] Liu Y, Ai K, Lu L (2014). Polydopamine and its derivative materials: synthesis and promising applications in energy, environmental, and biomedical fields. Chem Rev.

[22] Liu YL, Ai KL, Ji XY, Askhatova D, Du R, Lu LH, Shi JJ (2017). Comprehensive insights into the multi-antioxidative mechanisms of melanin nanoparticles and their application to protect brain from Injury in ischemic stroke. J Am Chem Soc.

[23] Nam J, Son S, Ochyl LJ, Kuai R, Schwendeman A, Moon JJ (2018). Chemo-photothermal therapy combination elicits anti-tumor immunity against advanced metastatic cancer. Nat Commun.

[24] Huang L, Liu M, Huang H, Wen Y, Zhang X, Wei Y (2018). Recent advances and progress on melanin-like materials and their Biomedical Applications. Biomacromolecules.

[25] Li J, Wang T, Kirtane AR, Shi Y, Jones A, Moussa Z, Lopes A, Collins J, Tamang SM, Hess K et al. Gastrointestinal synthetic epithelial linings. Sci Transl Med 2020; 12. 10.1126/scitranslmed.abc0441 PMC8221077 32848090

[26] Hu SS, Yang ZX, Wang S, Wang LP, He QQ, Tang H, Ji P, Chen T. Zwitterionic polydopamine modified nanoparticles as an efficient nanoplatform to overcome both the mucus and epithelial barriers. Chem Eng J 2022; 428.

[27] Poinard B, Kamaluddin S, Tan AQQ, Neoh KG, Kah JCY (2019). Polydopamine Coating enhances Mucopenetration and Cell Uptake of nanoparticles. ACS Appl Mater Interfaces.

[28] Li J, Hou W, Lin S, Wang L, Pan C, Wu F, Liu J (2022). Polydopamine nanoparticle-mediated dopaminergic immunoregulation in colitis. Adv Sci (Weinheim Ger).

[29] Wen X, Xi K, Tang Y, Bian J, Qin Y, Xiao W, Pan T, Cheng X, Ge Z, Cui W (2023). Immunized microspheres Engineered Hydrogel membrane for reprogramming macrophage and mucosal repair. Small.

[30] Gao S, Zheng H, Xu S, Kong J, Gao F, Wang Z, Li Y, Dai Z, Jiang X, Ding X, Lei H (2023). Novel Natural Carrier-Free Self-assembled nanoparticles for treatment of Ulcerative Colitis by Balancing Immune Microenvironment and Intestinal Barrier. Adv Healthc Mater.

[31] Yang F, Cheung PCK. Fungal β-Glucan-based nanotherapeutics: from fabrication to application. J Fungi 2023; 9. 10.3390/jof9040475 PMC10143420 37108930

[32] Xu J, Ma Q, Zhang Y, Fei Z, Sun Y, Fan Q, Liu B, Bai J, Yu Y, Chu J (2022). Yeast-derived nanoparticles remodel the immunosuppressive microenvironment in tumor and tumor-draining lymph nodes to suppress tumor growth. Nat Commun.

[33] Mo X, Sun Y, Liang X, Li L, Hu S, Xu Z, Liu S, Zhang Y, Li X, Liu L (2022). Insoluble yeast beta-glucan attenuates high-fat diet-induced obesity by regulating gut microbiota and its metabolites. Carbohydr Polym.

[34] Cao Y, Sun Y, Zou S, Duan B, Sun M, Xu X (2018). Yeast beta-glucan suppresses the chronic inflammation and improves the Microenvironment in adipose tissues of ob/ob mice. J Agric Food Chem.

[35] Sun Y, Shi X, Zheng X, Nie S, Xu X (2019). Inhibition of dextran sodium sulfate-induced colitis in mice by baker’s yeast polysaccharides. Carbohydr Polym.

[36] Zhao P, Xia X, Xu X, Leung KKC, Rai A, Deng Y, Yang B, Lai H, Peng X, Shi P (2021). Nanoparticle-assembled bioadhesive coacervate coating with prolonged gastrointestinal retention for inflammatory bowel disease therapy. Nat Commun.

[37] Zhou J, Li M, Chen Q, Li X, Chen L, Dong Z, Zhu W, Yang Y, Liu Z, Chen Q (2022). Programmable probiotics modulate inflammation and gut microbiota for inflammatory bowel disease treatment after effective oral delivery. Nat Commun.

[38] Wan Y, Yang L, Jiang S, Qian D, Duan J (2022). Excessive apoptosis in Ulcerative Colitis: Crosstalk between apoptosis, ROS, ER stress, and intestinal homeostasis. Inflamm Bowel Dis.

[39] Dong L, Xie J, Wang Y, Jiang H, Chen K, Li D, Wang J, Liu Y, He J, Zhou J (2022). Mannose ameliorates experimental colitis by protecting intestinal barrier integrity. Nat Commun.

[40] Mudter J, Neurath MF (2007). Il-6 signaling in inflammatory bowel disease: pathophysiological role and clinical relevance. Inflamm Bowel Dis.

[41] Ma L, Ni Y, Wang Z, Tu W, Ni L, Zhuge F, Zheng A, Hu L, Zhao Y, Zheng L, Fu Z (2020). Spermidine improves gut barrier integrity and gut microbiota function in diet-induced obese mice. Gut Microbes.

[42] Sun S, Luo L, Liang W, Yin Q, Guo J, Rush AM, Lv Z, Liang Q, Fischbach MA, Sonnenburg JL (2020). Bifidobacterium alters the gut microbiota and modulates the functional metabolism of T regulatory cells in the context of immune checkpoint blockade. Proc Natl Acad Sci USA.

[43] Mazzarella G, Perna A, Marano A, Lucariello A, Rotondi Aufiero V, Sorrentino A, Melina R, Guerra G, Taccone FS, Iaquinto G, De Luca A (2017). Pathogenic role of Associated Adherent-Invasive Escherichia coli in Crohn’s Disease. J Cell Physiol.

[44] Mata-Garrido J, Xiang Y, Chang-Marchand Y, Reisacher C, Ageron E, Guerrera IC, Casafont I, Bruneau A, Cherbuy C, Treton X (2022). The heterochromatin protein 1 is a regulator in RNA splicing precision deficient in ulcerative colitis. Nat Commun.

[45] Wu C, Chu B, Kuang L, Meng B, Wang X, Tang S (2013). Synthesis of beta-1,3-glucan esters showing nanosphere formation. Carbohydr Polym.

