# Supplementary material for: Attenuation of inflammatory bowel disease by oral administration of mucoadhesive polydopamine-coated yeast β-glucan via ROS scavenging and gut microbiota regulation
Source: J Nanobiotechnology. 2024 Apr 12;22:166. doi: 10.1186/s12951-024-02434-3 (PMC11010398; doi:10.1186/s12951-024-02434-3)
Supplement: Supplementary file 1 — Supplementary Material 1 [file 12951_2024_2434_MOESM1_ESM.docx]

**Additional File 1**

**Supplementary Information**

**Attenuation of Inflammatory Bowel Disease by Oral Administration of Mucoadhesive Polydopamine-Coated Yeast β-glucan via ROS Scavenging and Gut Microbiota Regulation**

Fan Yang^a^, Yuting Su^a^, Chi Yan^a^, Tianfeng Chen^b^, Peter Chi Keung Cheung ^a*^

^a^ *School of Life Sciences, The Chinese University of Hong Kong, Shatin, New Territories, Hong Kong, China*

*^b^College of Chemistry and Materials Science, Jinan University, Guangzhou, China*

*Corresponding author: Peter Chi Keung Cheung

E-mail: [petercheung@cuhk.edu.hk](mailto:petercheung@cuhk.edu.hk)


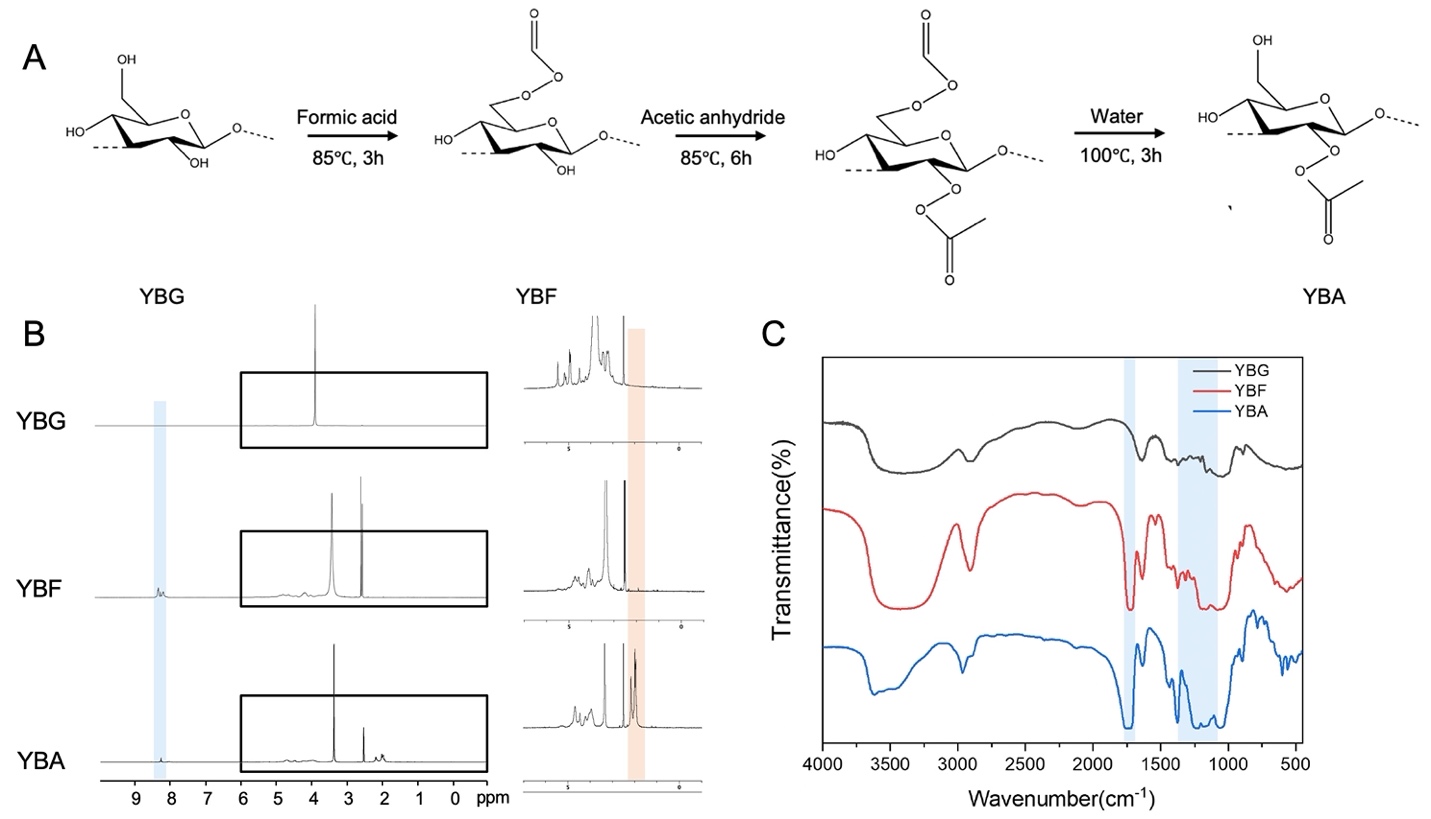


**Fig. S1**. Preparation of YBNs. (A) Synthesis route of yeast β-glucan formate (YBF) and yeast β-glucan acetate (YBA). (B) ^1^H NMR spectra for yeast β-glucan (YBG), YBF and YBA. (C) FT-IR spectrum of YBG, YBF and YBA.


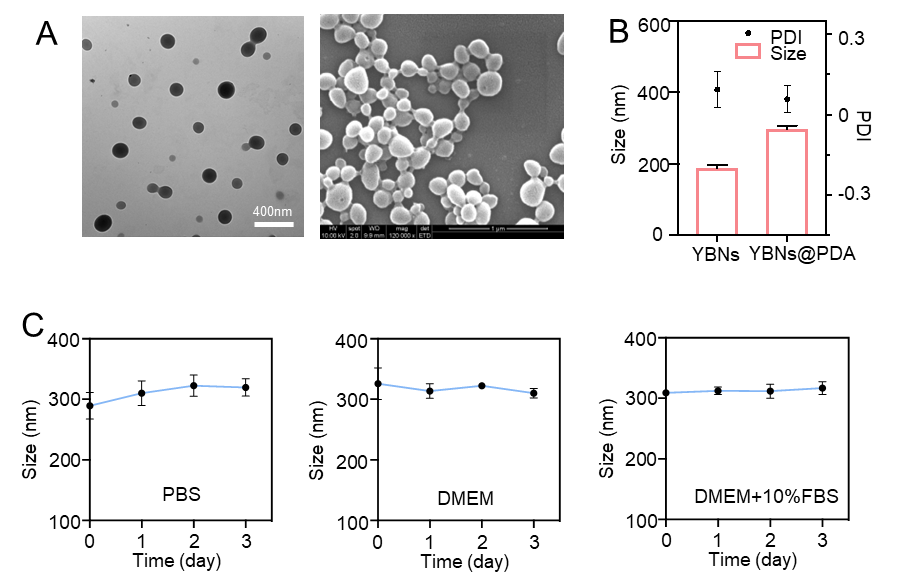


**Fig. S2**. Preparation and characterization of YBNs@PDA and YBNs. (A) TEM and SEM images of YBNs. (B) Size distribution and PDI of YBNs and YBNs@PDA. (C) Hydrodynamic diameter changes of YBNs@PDA in PBS, DMEM and DMEM+10% FBS, respectively.


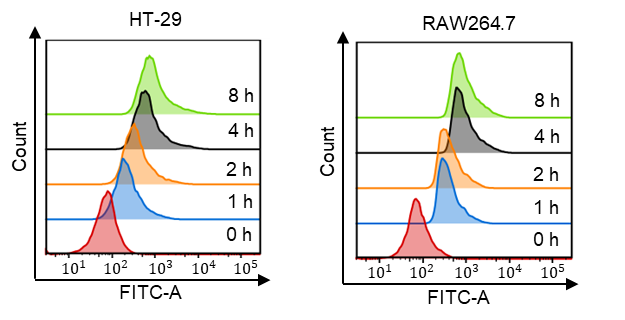


**Fig. S3**. Flow cytometry analysis of FITC-labeled YBNs@PDA after incubation with HT-29 or RAW264.7 cells for different time.


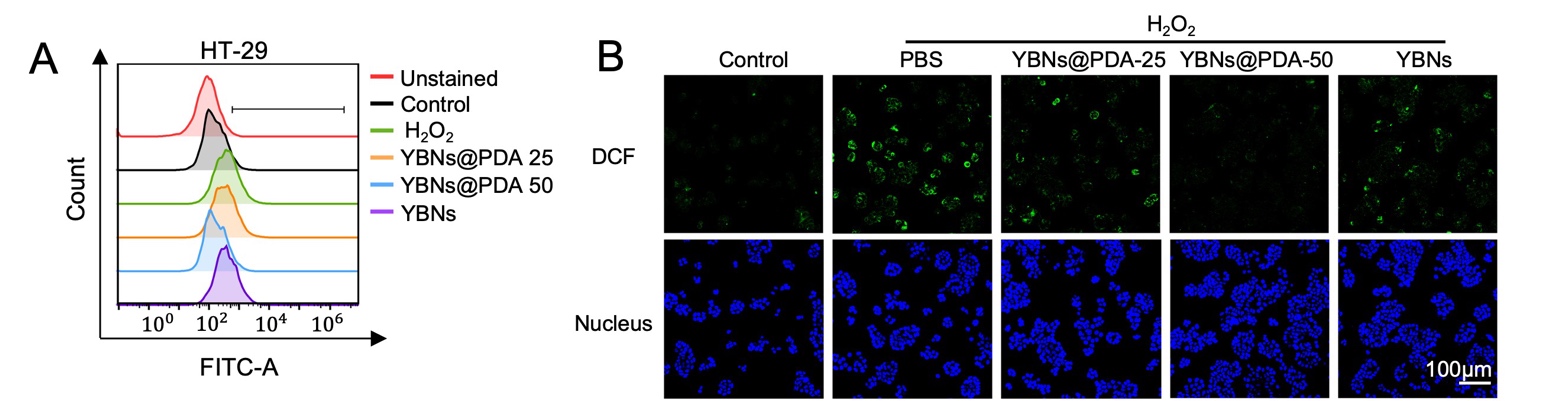


**Fig. S4**. Flow cytometry analysis (A) and CLSM images (B) of intracellular ROS levels of HT-29 cells after different treatments.


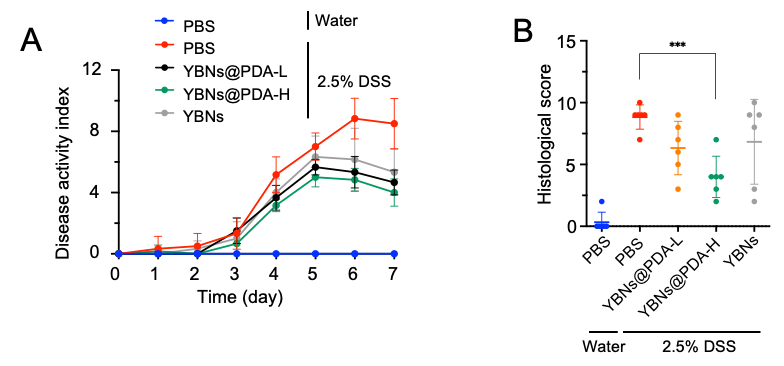


**Fig. S5**. Protective effect of YBNs@PDA in a DSS-induced acute colitis mice model (A) Disease activity index (DAI) score was recorded and analyzed. (B) Histological damage scores of colon tissue were measured. Data are presented as mean ± standard deviation from a representative experiment (n = 6).


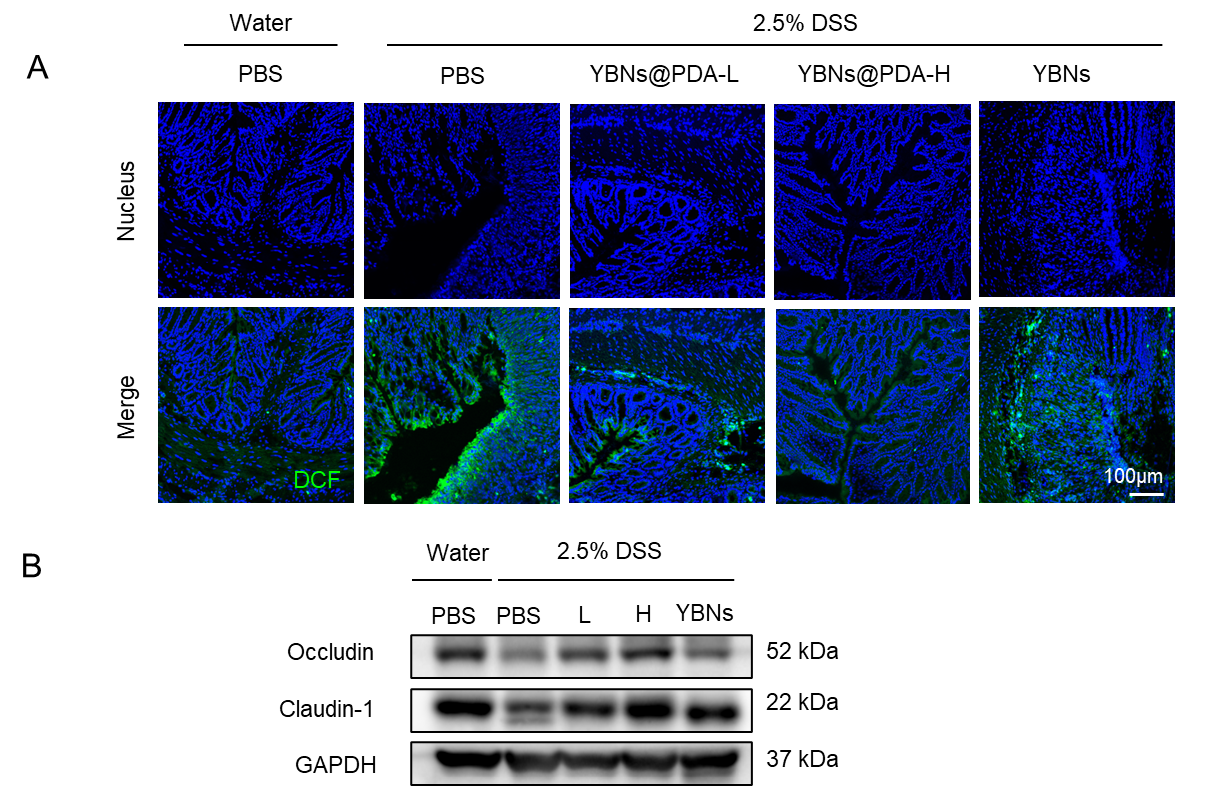


**Fig. S6**. (A) DCF staining of colon tissue to measure ROS level. (B) Western blot analysis of occludin and claudin-1 proteins in mice colon tissues. (L: 5 mg/kg YBNs@PDA; H: 10 mg/kg YBNs@PDA).


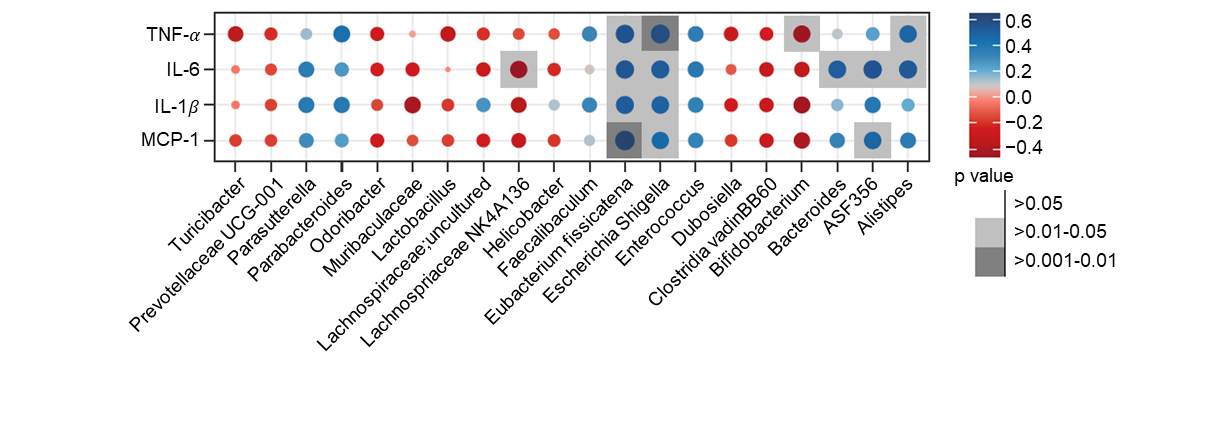


**Fig. S7**. Correlation analysis between colitis-related indices and specific gut microbiota composition at genus level.


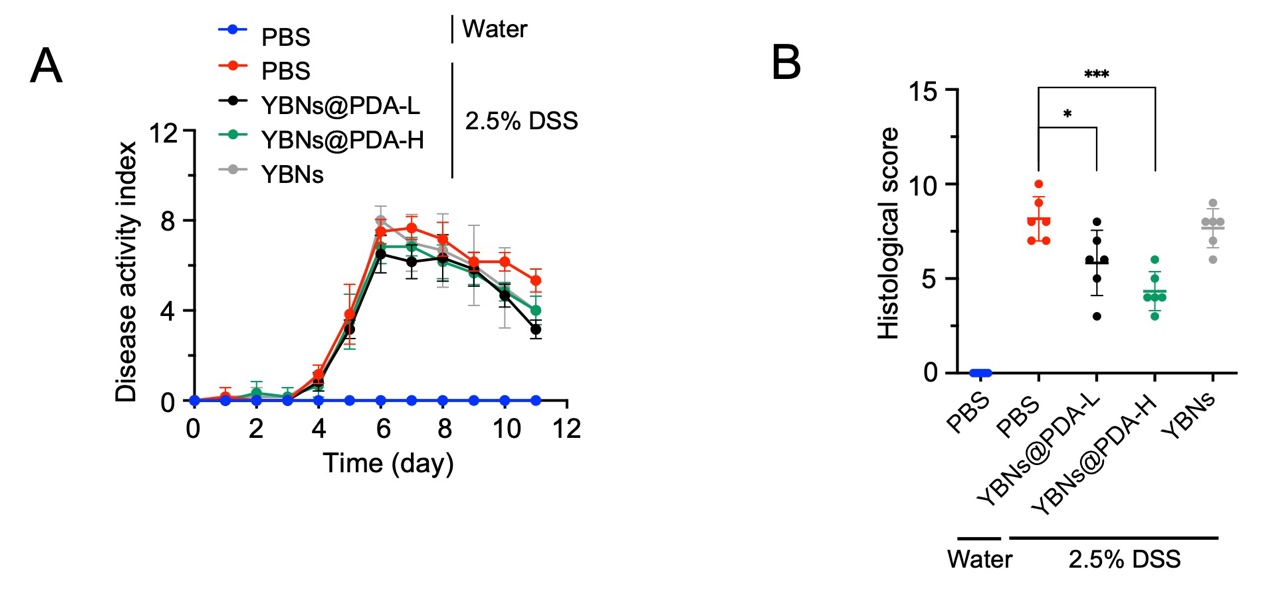


**Fig. S8**. Colitis alleviation effect of YBNs@PDA in a delayed therapeutic setting. (A) Disease activity index (DAI) score was recorded and analyzed. (B) Histological damage scores of colon tissue were measured (L: low dose: 5 mg/kg; H: High dose: 10mg/kg). Data are presented as mean ± standard deviation from a representative experiment (n = 6).


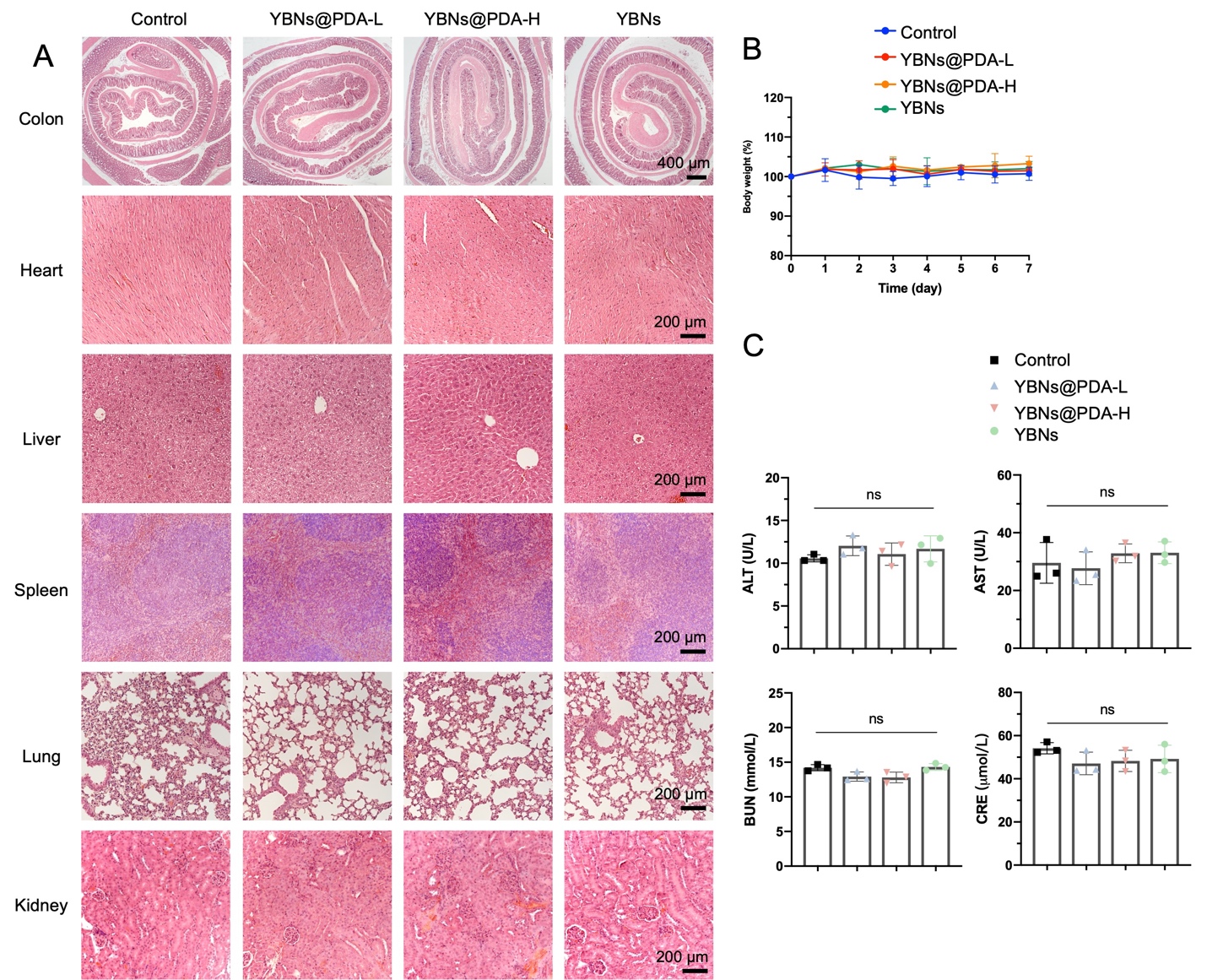


**Fig. S9**. Biocompatibility testing of YBNs and YBNs@PDA. (A) Histological images of H&E-stained major organ slices harvested from the mice after PBS, YBNs (10mg/kg) or YBNs@PDA (Low dose: 5 mg/kg; High dose: 10mg/kg) treatments. (B) Body weight changes of mice in each group. (C) ALT, AST, BUN and CRE levels in the serum of mice in each group. Data are presented as mean ± standard deviation from a representative experiment (n = 3). ns means not significant.
